# Microbial Inulinases: Characterization, Properties, and Potential Contribution to Metabolic and Nutritional Health

**DOI:** 10.3390/molecules31142519

**Published:** 2026-07-19

**Authors:** Anca Daniela Raiciu, Mihaela-Carmen Eremia, Mariana Gratiela Vladu, Maria-Monica Petrescu, Dana-Maria Miu, Gabriela Valeria Săvoiu, Fawzia Sha’at, Ramona-Daniela Pavaloiu

**Affiliations:** 1 Department of Pharmacognosy, Phytochemistry, and Phytotherapy, Faculty of Pharmacy, Titu Maiorescu University, Gh. Şincai Street No.16, District 4, 040314 Bucharest, Romania; daniela_raiciu@yahoo.com; 2 Hofigal Export-Import S.A., Intrarea Serelor Street No.2, District 4, 042124 Bucharest, Romania; 3 National Institute for Chemical-Pharmaceutical Research and Development (ICCF), 112 Vitan Av., District 3, 031299 Bucharest, Romania; marianagratielavladu@gmail.com (M.G.V.); maria.m.petrescu@gmail.com (M.-M.P.); dana.miu92@gmail.com (D.-M.M.); gabriela.savoiu@gmail.com (G.V.S.); fawzya.shaat@gmail.com (F.S.); pavaloiu.daniella@gmail.com (R.-D.P.)

**Keywords:** microbial inulinase, inulin, FOS, prebiotics, metabolic diseases

## Abstract

Microbial inulinases are enzymes produced by bacteria, yeasts, and fungi that can hydrolyze inulin into fructose and fructooligosaccharides (FOSs). The article discusses the various types of inulinases (exo- and endo-inulinases), microbial sources, biochemical properties, and optimal activity conditions, which are critical for their use in biotechnological and food processes. Special emphasis is placed on inulin degradation products, particularly FOSs, which are known for their prebiotic properties. They promote the growth of beneficial intestinal microbiota, helping to maintain digestive health and improve nutrient absorption. Compounds produced by inulinases may play an important role in the prevention of metabolic diseases such as obesity, type 2 diabetes mellitus, and dyslipidemias by modulating the microbiota and regulating energy metabolism. In conclusion, microbial inulinases are a promising biotechnological tool for developing nutritional strategies to prevent metabolic diseases and improve overall health. Recent evidence demonstrates that advances in recombinant expression systems, enzyme engineering, immobilization technologies, and microbiome research have substantially expanded the industrial and biomedical potential of microbial inulinases. This review highlights emerging trends toward sustainable enzyme production, precision nutrition, and microbiome-targeted functional foods while identifying current limitations and future research priorities.

## 1. Introduction

The consumption of fructans has increased dramatically in recent years, as fructan-containing foods have become more common in modern diets such as plant-based, Mediterranean, and high-fiber diets. Increased fructan consumption has been associated with gastrointestinal symptoms, including cramps, flatulence, and bloating. As a result, dietary fibers—some of which are clinically proven prebiotics (such as inulin)—can cause gastrointestinal issues, despite their numerous benefits. Dietary consumption of fermentable, digestion-resistant prebiotics has been linked to a lower risk of cardiovascular disease, type 2 diabetes, and certain types of cancer, as well as promoting the growth of beneficial gut bacteria [1,2,3,4]. Gastrointestinal symptoms after consumption of fructan-rich foods may be associated with fructan intolerance or irritable bowel syndrome (IBS). Fructan sensitivity is thought to affect approximately 24% of IBS patients. Over the last decade, increased gastrointestinal symptoms, food intolerance, and food sensitivity to specific fruits, vegetables, beans, grains, and plant-based foods have been linked to dietary fermentable oligo-, di-, monosaccharides, and polyols (a class of compounds known as FODMAPs) in both people with irritable bowel syndrome and those in good health. Food intolerance and sensitivity occur through non-immune-mediated mechanisms that differ from food allergies [5,6,7].

In this context, emerging nutritional strategies include the use of prebiotics, particularly inulin, whose efficacy depends on its enzymatic breakdown catalyzed by inulinase, together with approaches aimed at modulating the intestinal microbiota.

Modulating the intestinal microbiota may improve nutrient absorption and support overall host health, as increasing evidence suggests that the gut microbiome influences immune regulation, endocrine signaling, and multiple physiological processes [8,9].

Digestive health research teams (for example, Bio-Cat and Casa de Santé) hypothesized that microbial inulinase supplementation may promote dietary fructan digestion in the stomach by breaking it down into fructose that can be absorbed by the upper intestine rather than fermenting in the lower intestine, resulting in the aforementioned digestive symptoms [10,11], due to their main characteristic. Inulinases have the specificity of hydrolyzing the β-2,1-D-fructosidic bonds in inulin, converting fructan inulin into pure fructose (high-fructose syrup) or fructooligosaccharides (FOSs) [12,13,14].

The fructan inulin, largely derived from plants such as chicory, ginger, garlic, or onion, has unique structural and physiological characteristics due to its β(2,1) bonds, which human digestive amylases cannot break down [15,16]. Due to ineffective microbial breakdown, its metabolic effects may be limited to an osmotic effect once it reaches the colon [17]. To maximize the metabolic activation of this prebiotic, an emerging nutritional strategy suggests either administering probiotic strains that produce inulinase as synbiotics or using microbial inulinases to pre-process inulin into fructo-oligosaccharides (FOSs) [10,18]. Thus, to test the hydrolysis capacity of an inulinase preparation (BIO-CAT, Inc.; Troy, VA, USA, standardized at no less than 2000 units of activity), Guice et al. (2023), using *Aspergillus tubingensis* (also known as *A. niger*), simulated gastric digestion under static conditions in vitro and concluded that the use of microbial inulinase as an exogenous enzyme supplement can reduce the effects of dietary fructan-type FODMAP exposure [10].

Initially isolated from the roots of *Helianthus tuberosus*, inulinases can be obtained from microbial, plant, and animal sources, but due to low extraction yields, animal and plant sources cannot be explored commercially [19]. In the current context of the evolution of biotechnology, microorganisms are the preferred sources for the production of inulinases because they are relatively easy to cultivate on specific media and produce significant amounts of enzymes [20].

As a result, the scientific community is interested in microbial inulinase not only because it aids in the production of high-purity fructose syrups but also because of its ability to produce fructo-oligosaccharides [13,21,22,23]. These short molecules are potent prebiotics that selectively stimulate the growth of beneficial bacteria (*Bifidobacterium* and *Lactobacillus*), thereby strengthening the intestinal barrier and lowering low-grade systemic inflammation, both of which contribute to insulin resistance [1,16,24,25].

Furthermore, the prevalence of metabolic diseases, such as obesity, non-alcoholic fatty liver disease, and type 2 diabetes, has alarmingly increased globally in recent decades [26]. 

The importance of product formulation for food enzyme supplementation is highlighted in the current scientific context, showing the need for a strict selection algorithm based on efficacy and scientific evidence. The use of liposomal nanosystems containing microbial inulinase is now being investigated for the aforementioned medicinal applications, which could provide potential therapeutic applications. Such formulations may represent potential alternatives to current formulas, potentially contributing to the development of products designed for the prevention and treatment of digestive diseases while also incorporating a new health benefit orientation [10].

In addition to those mentioned, inulinase can also be used to produce bioethanol [27], single-cell oils and proteins [28], citric acid [29], gluconic acid, lactic acid [30], butanol, sorbitol, and so on [13,20].

New trends in inulinase research focus on the development of sustainable biotechnologies and the bioeconomy [2,31,32,33,34].

These studies investigate microorganisms from extreme or marine environments in order to isolate enzymes with superior thermal and operational stability, as well as use molecular dynamics simulations and molecular docking to better understand catalytic mechanisms and develop modified thermophilic variants [35,36,37,38].

Optimization of microbial biosynthesis can be achieved through a complete valorization of renewable resources and the transformation of agricultural waste into value-added products. To increase the reuse and lifetime of the biocatalyst in industrial processes, the integration of inulinases into complex systems is achieved by optimizing enzyme immobilization methods (for example, on economical supports such as eggshells) [39,40,41,42,43,44].

Although several reviews have summarized the biochemical characteristics, production methods, and industrial applications of microbial inulinases, recent advances in microbiome research, enzyme engineering, sustainable bioprocesses, and functional food development have considerably expanded the scientific interest in these enzymes. This review provides an updated and integrated perspective by critically discussing recent developments in microbial inulinase production, enzyme engineering, their role in generating bioactive fructo-oligosaccharides, and the potential implications of these advances for metabolic and nutritional health.

## 2. Characterization of Microbial Inulinases

Microbial inulinases are of considerable interest for both basic research and industrial applications. Various microorganisms produce β-fructofuranosidases 2,1-β-D-fructan (inulinases), and their synthesis is influenced by growth conditions, particularly the type of carbon source used. The biophysical and biochemical properties of the inulinases that have been studied thus far vary. These enzymes break down β-(2,1) bonds in the inulin chain to produce fructose and fructo-oligosaccharide (FOS) units. The synthesis and purification of microbial inulinase have advanced significantly in recent years. Genetic engineering can also improve enzyme synthesis and its functional characteristics. The literature provides information on molecular weight as well as how temperature, pH, and metal ions affect inulinase activity and stability [14,20,29,45,46]. Certainly, the enzyme’s response to these variables is primarily determined by the producing strain.

### 2.1. Structural and Functional Features of Inulinases

Inulinases are a significant class of hydrolytic enzymes that belong to the glycoside hydrolase family 32 (GH32). This family of enzymes includes fructan-hydrolases (exo-inulinases, endo-inulinases, and levanases); fructosyl-transferases, which help with the metabolism of complex carbohydrates; and invertases, which convert sucrose into glucose and fructose [47,48,49].

In comparison to invertase, inulinases can be characterized by their hydrolytic action and substrate specificity for inulin. Inulin is a linear vegetal polymer with a glucose group at the reducing end connected by an α-D-1,2 glycosidic connection and fructose monomeric units joined by β-D-2,1 glycosidic bonds [50,51]. After starch, it is the second most abundant vegetable polysaccharide found in nature and is naturally produced by many plant species belonging to the families *Liliaceae*, *Asteraceae*, *Poaceae*, *Campanulaceae*, etc. Inulin is found in many common plants, such as dahlia, chicory, leek, garlic, asparagus, and dandelion [52,53,54]. 

**Figure 1:**
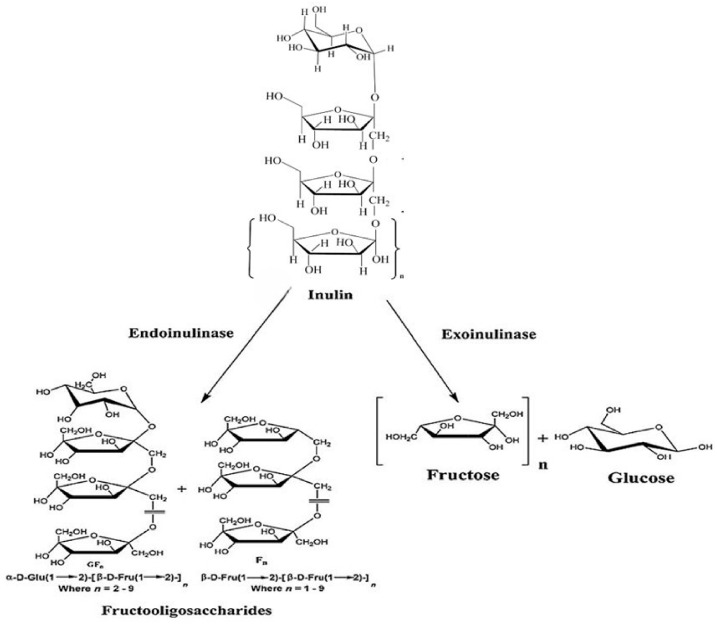
Mode of action of inulinases on inulin.

The primary end products of inulinases’ hydrolytic action on inulin are fructose or fructooligosaccharides. Inulinases are classified as exo-inulinases (β-D-fructan fructohydrolase; EC 3.2.1.80) and endo-inulinases (1-β-D-fructan fructanohydrolase; EC 3.2.1.7) based on how they interact with inulin. Exo-inulinases produce fructose by cleaving successive terminal units from the non-reducing end of the inulin molecule. Exo-inulinases hydrolyze not only inulin but also sucrose and the remaining fructose from raffinose. In contrast, endo-inulinases are specific for inulin and act on the internal β-2,1 glycosidic bonds of inulin that are located far from the ends of the polymer chain and produce fructooligosaccharides by breaking the bonds between fructose units (Figure 1) [13,33,53,54].

The property of having exo- or endo-action depends on the microbial origin of the enzyme. Research has shown that inulinases produced by fungi are generally exo-inulinases. Some strains, like *Aspergillus ficuum*, produce both endo- and exo-inulinases, resulting in a more efficient conversion of inulin to fructose [55].

#### 2.1.1. Molecular Mass of Inulinases

The molecular weights of inulinases vary in the range of 28–450 kDa (Table 1) [56,57]. Thus, Sheng et al. purified and characterized inulinase produced by *Cryptococcus aureus*, and its molecular weight was estimated at 60.0 kDa [58]. For *Pichia guilliermondii*, the obtained inulinases have a molecular weight of 50 kDa [59], and inulinase produced by *Kluyveromyces fragilis* has a molecular weight of 250 kDa [60].

The yeast *Kluyveromyces* species produces three forms of inulinase with molecular weights of 42, 65, and 57 kDa. The enzyme produced from *Kluyveromyces marxianus* DSM 70,106 was found to be a dimer with a molecular weight of 200 kDa [61,62,63].

The molecular mass of inulinases produced by bacterial strains was similar to those produced by yeasts. Kassem et al. characterized inulinases produced by *Arthrobacter* spp. and estimated a molecular weight of 75 kDa [64].

The molecular mass of inulinases produced by fungal strains was estimated between 50 kDa and 300 kDa, namely, *Aspergillus ochraceus*—79 kDa [65], *Penicillium* spp.—68 kDa [66,67], and *Fusarium oxysporum*—300 kDa [68].

**Table 1:** Molecular weights of microbial inulinases.

Producer Microorganism	Molecular Weight, kDa			Reference
	Gel-Chromatography Method	SDS-PAGE Electrophoresis Method	Method Is Not Specified	
*Candida kutaonensis* sp. nov. KRF1T		55	57.3 ^b^	[69]
*Cryptococcus aureus* G7a		60		[58]
*Kluyveromyces* species Y-85		60	59.5 ^b^	[70]
*Kluyveromyces fragilis*			250	[60]
*Kluyveromyces marxianus*			72	[71]
*Kluyveromyces marxianus* var. *bulgaricus* ATCC16045	77	57		[72]
*Kluyveromyces marxianus* DSM 70106	200			[63]
*Kluyveromyces marxianus* Y-303	63	54.8 and 8.4		[61]
*Pichia guilliermondii*			50	[59]
*Saccharomyces* sp.W0			57.8 ^b^	[73]
*Yarrowia lipolytica* ^a^		78.9	82.4 ^b^	[74]
*Arthrobacter species* HJ7				[64,75]
*Aspergillus* species			56 ^b^	[76]
*Aspergillus awamori*	87.7	76.9 and 10.1		[77]
*Aspergillus awamori* var. 2250		69.63 ^c^		[78]
*Aspergillus ficuum*		63	63 ^c^	[79]
*Aspergillus ficuum*	54			[80]
*Aspergillus niger*	50	89	57 ^d^	[81]
*Aspergillus niger* 12		81	57.3 ^b^	[82]
*Aspergillus terreus* CCT 4083	56	57		[83]
*Fusarium oxysporum*			300	[68]
*Penicillium* species			83, 66, 63	[66,67]
*Streptomyces* sp.			45	[84]
*Xanthomonas oryzae* No. 5			139	[85]

^a^ Inulinase was expressed in this organism. ^b^ Molecular weights calculated basing on the primary structure. ^c^ After deglycosylation. ^d^ Molecular weight calculated using mass spectrometry method (MALDI-TOF MS).

Although the microorganisms presented in Table 1 differ considerably in their taxonomy and ecological niches, they all represent valuable biological sources of inulinases with distinct catalytic properties. Beyond their industrial relevance, microbial inulinases play an important indirect role in the production of fructo-oligosaccharides (FOSs), which are recognized as functional food ingredients capable of selectively stimulating the growth of beneficial intestinal microorganisms, including *Bifidobacterium* and *Lactobacillus* species. Consequently, the selection of appropriate microbial producers is not only important for enzyme production efficiency but also for the development of sustainable biotechnological processes aimed at obtaining prebiotic compounds with potential applications in gut microbiota modulation and metabolic health.

#### 2.1.2. Molecular Structure of Microbial Inulinases

To understand the mechanism of inulinase activity in vivo, structural and functional properties, as well as the enzyme’s molecular and supramolecular organization, must be investigated.

**Figure 2:**
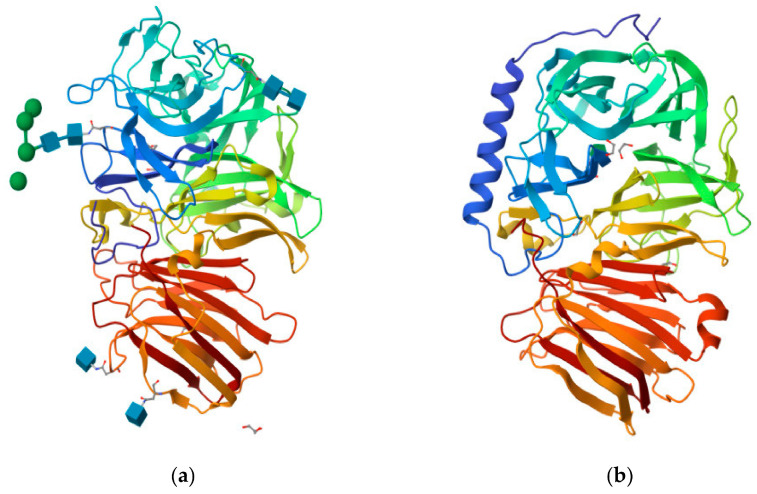
Structures of microbial inulinases: (**a**) Crystal structure of hydrolase exo-inulinase from *Aspergillus awamori*. (**b**) Crystal structure of hydrolase endo-inulinase from *Bifidobacterium adolescentis* expressed in *Escherichia coli* [86,87].

A key factor in the catalytic activity of inulinases is their supramolecular organization, which comprises their three-dimensional architecture, aggregation forms (dimers and tetramers), and interactions with the microenvironment. This organization directly influences the efficiency of inulin conversion into fructose or fructooligosaccharides. Most inulinases (exo- and endo-) have a characteristic bimodular structure, GH32, organized into two distinct functional domains [14,86]. The catalytic domain is responsible for binding the substrate (e.g., inulin) and breaking it down into fructose and fructo-oligosaccharides, while the secondary domain helps anchor the enzyme to the inulin polymer, increasing the enzyme’s catalytic efficiency (Figure 2).

The two inulinase structures consist mainly of around 518 amino acids in their sequences. Because of differences in expression between endo- and exo-inulinases, further studies are required to clarify whether structural differences are responsible for inulinase function [86,87].

Although microbial inulinases share the conserved GH32 catalytic framework, subtle structural variations within the substrate-binding pocket largely determine their catalytic behavior and substrate specificity. In particular, differences in the length and flexibility of surface loops surrounding the catalytic cavity influence substrate accessibility and explain the distinct hydrolytic patterns of exo- and endo-inulinases. Exo-inulinases possess a relatively narrow catalytic pocket that favors the sequential removal of terminal fructose residues, whereas endo-inulinases contain a more open catalytic cleft capable of accommodating internal regions of the inulin polymer, resulting in the production of fructo-oligosaccharides. These structure–function relationships are increasingly being explored through X-ray crystallography, molecular docking, and molecular dynamics simulations, providing valuable information for the rational engineering of enzymes with improved thermos-stability, substrate specificity, and catalytic efficiency [37,38].

The three-dimensional structure consists of an N-terminal domain containing the active site, forming a 5-bladed β-propeller, and a C-terminal domain with two β-sheets, forming a β-sandwich module. The predominance of β-sheet structures contributes to enzyme stability. Exo-inulinases are often more stable than endo-inulinases [13,88,89].

Inulinases function through an anomeric configuration retention mechanism (“*anomeric retention*”), being enzymes of the type “*retaining*”, which use two carboxylic acids (usually aspartate or glutamate) in the active site. The RDP (Arginine–Aspartic Acid–Proline) motif, crucial for inulin identification, is a highly conserved structural characteristic seen in inulinases (both endo- and exo-inulases) [14,90].

Studies indicate that inulinases do not exclusively operate as monomers; they can also assemble into higher-order structures, including dimerization or aggregation, resulting in tertiary structures such as dimers or tetramers [68,69]. It has been shown that enzymes such as fructosyl transferase (1-SST) from *Aspergillus foetidus* (180 kDa) [71] and invertase (SUC2) from *Aspergillus niger* (210–240 kDa) function as dimers [91,92].

**Figure 3:**
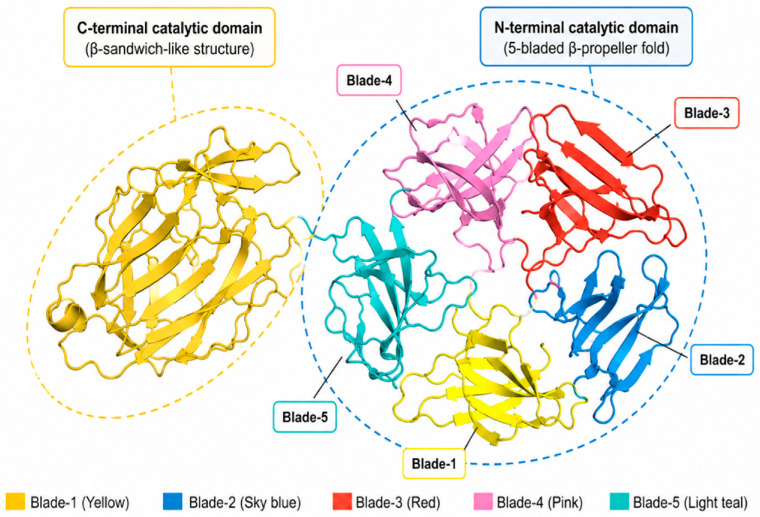
Representation of the three-dimensional structure of exo-inulinase from *Aspergillus awamori* (PDB ID: 1Y9G). The enzyme consists of an N-terminal catalytic domain with a five-bladed β-propeller fold (Blade-1 in yellow, Blade-2 in sky blue, Blade-3 in red, Blade-4 in pink, and Blade-5 in light teal) and a C-terminal catalytic domain with a β-sandwich-like structure [93].

Experimental studies on *Aspergillus niger* and *Penicillium* spp. have shown the presence of inulinase forms with apparently varying molecular masses (e.g., 54–86 kDa), suggesting various levels of quaternary organization. To date, three-dimensional structures have been reported [93], only for inulinases from *Aspergillus ficuum* and *Aspergillus awamori* (Figure 3).

The dimerisation of inulinases, such as those derived from *Aspergillus ficuum*, primarily involves interactions between monomers facilitated by nonpolar amino acid residues. In contrast, 1-FEHs (fructan 1-exohydrolases) have been observed to usually function as monomers [94], being important enzymes with potential applications in the pharmaceutical and food industries.

The structural differences that occur between exo- and endo-inulinases are due to, in the case of endo-inulinases, the presence of an additional pocket in the catalytic site, formed by two loops with the sequence W-M(I)-N-D(E)-P-N-G, which confers endo activity (breaking internal bonds). Exo-inulinases act by releasing the terminal fructose and are often more structurally stable and abundant in *Aspergillus* spp. (Figure 2).

Despite the considerable progress achieved in elucidating the three-dimensional structures of GH32 enzymes, only a limited number of microbial inulinases have been structurally characterized at high resolution. Consequently, important questions remain regarding the molecular determinants governing thermos-stability, catalytic efficiency, and substrate selectivity across different microbial species. Recent advances in computational enzyme engineering, including molecular dynamics simulations, machine learning-assisted protein design, and site-directed mutagenesis, are expected to accelerate the development of tailor-made inulinases with improved performance for industrial biotechnology and functional food production.

### 2.2. Structural Properties of Microbial Inulinases

It is known that endo-inulinase, exo-inulinase, invertase, levanase, and fructosyltransferase belong to the group of fructofuranosidases of the glycoside hydrolase family 32. The catalytic process of most glycoside hydrolases usually involves two acidic residues, one of which acts as a nucleophile and the other as a proton donor.

Endo-inulinase from *Arthrobacter*, exo-inulinase from *Aspergillus awamori*, fructosyl-transferase from *Aspergillus foetidus*, levanase from *Gluconacetobacter diazotrophicus*, inulinase from *Aspergillus ficuum*, and invertase from *Thermotoga maritima* were used to characterize the structural and functional properties of inulinases from various microbial sources. Following these studies, it was discovered that all enzymes have three common sequences in their primary structure: WMN(D/E)PN, RDP, and EC(P), which have special roles [81,89,95,96,97,98,99,100].

The WMN(D/E)PN sequence is located in the enzyme’s N-terminal region and is required for enzymatic activity. It contains residues that are critical for substrate binding and catalysis. The tryptophan (Trp), glutamate (Glu), or aspartate (Asp) residues in this sequence are required for the activity of endo-inulinases from *Aspergillus ficuum* (e.g., Trp17 and Glu20).

The RDP (Arginine–Aspartate–Proline) sequence, which is highly conserved in the GH32 family, is important for both stabilizing the transition state during hydrolysis and recognizing inulin. It is responsible for coordinating the acid–base catalytic role.

The EC(P) (Glutamate–Cysteine–(Proline)) sequence in the active site contains the glutamate residue (E), which acts as an acid–base catalyst in the hydrolysis mechanism. In various microorganisms, it can occur as ECP or ECV.

The active site of *Aspergillus awamori* inulinase consists of the carboxyl group of aspartic acid and the imidazole group of histidine. Inulinase’s carboxyl group forms a hydrogen bond with histidine’s imidazole group. The electrophile–nucleophile system of the carboxyl–imidazole interaction thus plays an important role in the hydrolysis of inulin. Exo-inulinases from *Aspergillus niger* and *Penicillium* species are thought to have aspartic and glutamic acid carboxyl groups in their active sites [87].

After N-bromosuccinimide modification, *Penicillium* species produce a completely inactive endo-inulinase, indicating that the active site contains at least one tryptophan residue. It is unclear what role Trp17 plays in endo-inulinase’s catalytic properties. It may form hydrogen bonds with the C6 hydroxyl group of the fructose ring as part of the inulin chain, ensuring that the substrate is properly oriented at the active site [95].

The glycoside hydrolases of the exo-inulinase and endo-inulinase categories differ in their activity, which is reflected in the architecture of their active sites. Table 2 and Table 3 present the identities of exo- and endo-inulinases obtained from various microorganisms in comparison with related enzymes.

A comparative study performed on the endo-inulinase obtained from *Aspergillus niger* and the exo-inulinase from *Bacillus stearothermophilus* shows that there are certain amino acids present on the surface of the enzymes, which establish a complex network of interactions with other residues of the active site of the enzyme, playing a major role in maintaining its conformation and contributing to the chemoselectivity of the enzyme [39,75,77,87,96,101].

The diversity of the microbial producers and production strategies summarized in Table 2 highlights the considerable biotechnological potential of inulinases. Many microorganisms, especially filamentous fungi, are used more as industrial enzyme producers than as probiotics. However, the enzymes they make are very important for making high-value functional ingredients. The enzymatic production of short-chain fructo-oligosaccharides offers important advantages over chemical synthesis, including higher specificity, lower environmental impact, and better preservation of bioactive compounds. Therefore, microbial inulinases constitute an important link between industrial biotechnology and the development of functional foods designed to promote intestinal health.

**Figure 4:**
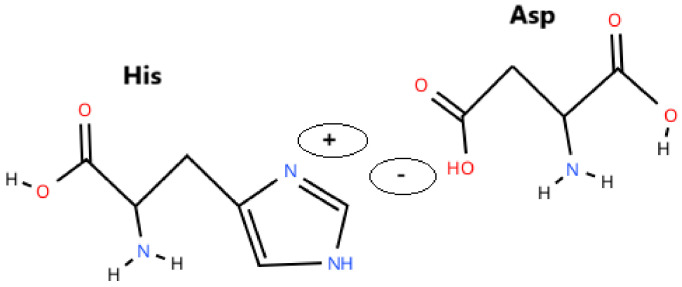
Simplified representation of the catalytic site of exo-inulinase. The catalytic mechanism is initiated by the interaction between the catalytic aspartate residue (Asp), acting as the nucleophile, and the histidine residue (His), which contributes to proton transfer and stabilization of the transition state during hydrolysis of β-(2 → 1)-fructosidic bonds. The catalytic arrangement promotes the sequential release of terminal fructose residues from the non-reducing end of the inulin chain.

**Figure 5:**
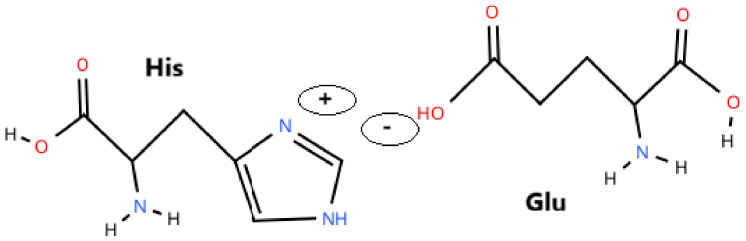
Simplified representation of the catalytic site of endo-inulinase. In contrast to exo-inulinases, the catalytic glutamate residue (Glu) facilitates the cleavage of internal β-(2 → 1)-fructosidic bonds within the inulin polymer. Structural differences in the catalytic pocket enable endo-inulinases to generate fructo-oligosaccharides (FOSs) instead of free fructose.

The main differences between the structures of *Aspergillus niger* endo-inulinase and *Bacillus stearothermophilus* exo-inulinase were found in the enzymes’ catalytic sites. The two inulinases have structural similarities, presenting an Asp residue (Asp24 in the *B. stearothermophilus* enzyme and Asp41 in *A. awamori*) that is thought to be responsible for nucleophilic attack (Figure 4) [87]. In the active site of endo-inulinase, Asp is replaced by Glu43 (Figure 5). The large size of the natural substrates causes the catalytic site of endo-inulinase to be quite large, accounting for approximately 90 of the 516 residues. In comparison, the active site of exo-inulinase, which acts on the terminal end of the polymer, is smaller and includes 42 of the total 493 amino acids [39].

**Table 2:** Amino acid sequences of exo-inulinases and related enzymes from various organisms.

Producer Microorganism	Enzyme for Comparing	Identity %	Reference
*Aspergillus niger*	Fructosyltransferase 1-SST from *Aspergillus foetidus*	100	[66,79,83,91,96,102,103]
	Exo-inulinase from *Aspergillus awamori*	91	
	Exo-inulinase from *Kluyveromyces marxianus*	38	
	Endo-inulinase from *Penicillium* species strain TN-88	37	
	Endo-inulinase from *Penicillium purpurogenum*	36	
	Endo-inulinase from *Aspergillus niger*	35	
	Levanase from *Bacillus subtilis*	42	
	Levanase from *Actinomyces naeslundii*	41	
	Levanase from *Bacillus polymyxa*	39	
	Invertase from *Saccharomyces cerevisiae*	40	
	Invertase from *Pichia anomala*	39	
*Aspergillus awamori*	Inulinase from *Bacillus subtilis*	13	[87,104]
	Levansucrase from *Bacillus subtilis*	13	
*Bacillus polymyx* MGL21	Exoinulinase from *Pseudomonas* *mucidolens*	57	[105]
	Exoinulinase from *Aspergillus niger*	39	
	Levanase from *Bacillus subtilis*	41	
*Escherichia coli* ^a^ BL21(DE3) (InuAMN8)	Levanase from *Arthrobacter phenanthrenivorans* Sphe3	81.1	[106]
	Exo-inulinase from *Bacillus* spp. snu-7	57.8	

^a^ Inulinase was expressed in this organism from *Arthrobacter* sp. MN8.

**Table 3:** Amino acid sequences of endo-inulinases and related enzymes from various organisms.

Producer Microorganism	Enzyme for Comparing	Identity %	Reference
*Aspergillus* sp.	Inulinase from *Aspergillus ficuum*	97	[79,82,107]
	Inulinase from *Aspergillus niger* inuA	96	
	Inulinase from *Aspergillus niger* inuB	96	
	Endo-inulinase from *Penicillium spurpurogenum*	69	
	Exo-inulinase from *Aspergillus niger*	33	
	Exo-inulinase from *Kluyveromyces maxianus*	22	
*Arthrobacter* species S37	Endo-inulinase from *Aspergillus niger*	15.3	[102,103,104,107,108]
	Endo-inulinase from *Aspergillus ficuum*	15.3	
	Endo-inulinase from *Aspergillus ficuum*	15.6	
	Exo-inulinase from *Kluyveromyces maxianus*	13.8	
	Levanase from *Bacillus subtilis*	16	
	Invertase from *Saccharomyces cerevisiae*	13.3	

The biochemical characteristics presented in Table 3 demonstrate the remarkable diversity of microbial inulinases regarding optimal pH, temperature, substrate specificity, and catalytic efficiency. These characteristics directly influence their suitability for different industrial and food-related applications. In particular, enzymes exhibiting high specificity and stability are increasingly considered attractive biocatalysts for the controlled production of fructo-oligosaccharides and other functional carbohydrates intended for incorporation into foods with prebiotic properties. Consequently, understanding these enzymatic characteristics contributes not only to process optimization but also to the development of functional ingredients capable of supporting a balanced intestinal microbiota.

The presence of the Arg-Asp-Pro (RDP) motif, which is also conserved in other enzyme classes such as fructosyl-transferases or invertases, is an intriguing common feature between the structures of endo-inulinase and exo-inulinase. This is a structural signature of family 32 glycoside hydrolases. This sequence is thought to be important for recognizing the pyranoside ring and has been linked to the enzyme’s specificity for the fructo-pyranoside residue [87].

The catalytic site of inulinase from *Aspergillus ficuum* differs from that of other inulinases. In the active site of endo-inulinases is a glutamate, while in other enzymes, it is an aspartate [81]. The replacement of the Thr100 residue, present in endo-inulinase, with a serine in other enzymes of the same family directly affects the catalytic capacity and substrate specificity. Although both threonine and serine have a hydroxyl group (-OH) that can form hydrogen bonds, the presence of a methyl group in the threonine structure increases its steric bulk and hydrophobicity slightly. In the case of endo-inulinases, this residue plays an essential role in properly positioning the inulin chain and stabilizing the transition state during cleavage of β-(2,1)-fructosidic bonds.

In addition, the Glu in the catalytic site is followed by a cysteine residue present in all inulinases except endo-inulinases, where it is substituted with a valine (Val234). This unique substitution of cysteine for valine in endo-inulinase plays an important structural role. This residue modifies the cavity volume and flexibility of the active site, obstructing chain-end binding and facilitating internal cleavage activity [80].

It has been noted that cysteine plays an essential role in the catalytic activity and structural stability of inulinases [14,57,109,110]. The specific roles of cysteine in their function include the transition state and the catalytic center. In the case of inulinases from Streptomyces strains with cysteine residues in the active site, the thiol group (-SH) forms a catalytic pair with other amino acids, such as histidine, responsible for breaking β-2,1-fructosidic bonds and stabilizing substrate transition states [111,112]. Cysteines that are not directly located in the active site are often responsible for the formation of disulfide bonds. These covalent bonds are crucial for maintaining the three-dimensional structure (β-propeller fold and β-sandwich domain) of inulinase, providing resistance against thermal stress and pH changes [14,113].

Taking into account the data from the specialized literature showing the dimensional characteristics of inulinases, it is found that there is no certainty about the existence of supramolecular organization in these enzymes. It is difficult to explain why an enzyme acts as a monomer in some organisms but not as a dimer or even a tetramer. Also, the practical significance of different degrees of glycosylation for inulinases from various microorganisms is still unclear.

### 2.3. Biochemical Characterization of Microbial Inulinases

The biochemical properties of inulinases are primarily dependent on their source; thus, inulinase production from various sources, including animals, plant tissues that store inulin, and multiple microorganisms, has been investigated [109]. Due to low enzymatic performance, commercial sources in plants and animals cannot be explored.

As a result, microbial sources for inulinase production have received increased attention due to their numerous benefits, including ease of use, culture, and genetic manipulation.

Due to the heat resistance of inulinase-producing bacterial strains, researchers have studied the production of these enzymes, which can be used at high temperatures in various industries [55,109].

The biochemical characterization of inulinases involves analyzing their structural, kinetic, and environmental parameters (pH and optimum temperature values) to determine their efficiency in inulin degradation.

#### 2.3.1. The pH Range and Optimal Temperature of Inulinase Activity

The pH optimal for inulinase activity isolated from fungi and yeasts ranges between 4.5 and 6.0 (Table 4). Inulinases produced by *Aspergillus niger* have an optimum pH of 4.4, while inulinases produced by *A. versicolor* have an optimum pH of 5.5, and *Penicillium janczewskii* produces inulinases with an optimum pH in the range of 4.8–5.0 [67]. Inulinases produced by *A. ochraceus* have optimal enzymatic activity at a pH of 4.5 [65].

There are inulinases with activity at acidic pH; for example, inulinases produced by *Kluyveromyces marxianus* produce inulinases with the highest activity at 3.5 [104], followed by *Pichia guilliermondii* at 3.4 [35].

In contrast, inulinases produced by some bacterial strains, such as *Arthrobacter* spp. [64] and *Bacillus polymyxa* [97,114], hydrolyze inulin at an optimal pH of 7.0–7.5.

Purified inulinases are usually most active between 50 and 60 °C. *Kluyveromyces* spp. strains produce inulinases with maximum activity in the range of 50–55 °C [115], whereas inulinases produced by *Pichia guilliermondii* [57,59], *A. ochraceus* [43], and *Streptomyces* spp. [116] have optimal activity and very good stability at 60 °C. *Fusarium oxysporum* [68], *Penicillium janczewskii* [67], and *A*. *niger* [88] produce inulinases with optimal activity in the range of 30–40 °C, indicating that the optimal temperatures of inulinases produced by different species of microorganisms are significantly different.

The biochemical diversity of microbial inulinases summarized in Table 4 illustrates the remarkable adaptability of these enzymes to different industrial and food-processing conditions. Variations in optimal pH, temperature, and stability determine their suitability for specific applications, including the enzymatic production of fructo-oligosaccharides (FOSs) and high-fructose syrups. From the perspective of functional foods, these enzymatic characteristics are particularly important because they influence the efficiency of FOS production under food-compatible processing conditions. Although many of the microorganisms listed, especially filamentous fungi, are exploited primarily as industrial enzyme producers rather than as probiotics, the inulinases they produce contribute indirectly to human health by enabling the sustainable production of prebiotic carbohydrates. These prebiotic compounds selectively stimulate beneficial gut microorganisms, such as *Bifidobacterium* and *Lactobacillus* spp., thereby linking microbial inulinases with gut microbiota modulation and the development of functional food ingredients.

**Table 4:** Properties of some microbial inulinases from various organisms.

Producer Microorganism	Molecular Mass (kDa)	Optimal pH	Optimum Temperature (°C)	pH Stability Range	Temperature Stability (°C)	Reference
*Kluyveromyces marxianus*	-	4.4	50	-	-	[115]
*Kluyveromyces fragilis*	250	-	55	-	-	[60]
*Yarrowia lipolitica*	-	4.5	50	3–7	50	[20]
*Pichia guilliermondii*	50	6.0	60	6–7	60	[46]
*Cryptococcus aureus*	60	5.0	50	4.0–6.5	65	[59]
*Arthrobacter* spp.	75	7.5	50	5–10.5	30–40	[64]
*Bacillus* spp.	-	7.0	-	6–8	25–40	[114]
*Streptomyces* spp.	-	6.0	60	-	60–70	[84]
*Aspergillus niger*	70	5.0	40	-	-	[107]
*Penicillium janczewskii*	-	4.8–5.0	35–45	-	-	[67]
*Aspergillus ochraceus*	79	4.5	60	-	60	[65]
*Fusarium oxysporum*	300	5.8–6.2	30–37	-	-	[68]

The enzymes Inu2 and Inu3 produced by *Rhizopus oligosporus* strain NRRL 2710 had the same optimum pH of 5.0 and optimum temperatures of 50 and 60 °C, respectively, as well as thermal stability up to 60 and 70 °C for 1 h and a high affinity for inulin. The enzyme activity was not significantly influenced by Mg^2+^, Ca^2+^, Zn^2+^, and EDTA, while Ni^2+^, Cu^2+^, Fe^2+^, and Co^2+^ showed a partial inhibitory effect, and Hg^2+^ had a strong inhibitory effect [111]. Gill et al. also observed that purification and immobilization of inulinase produced by *Aspergillus fumigatus* (MTCC no. 3009) on various supports improved its stability (it retained approximately 70% of its activity after 48 h at 60 °C) and half-life (22, 35, and 45 days for QAE-Sephadex, chitin, and ConA packed bed column reactors, respectively) [117].

The main focus of research on thermostable inulinases is their potential application in the industrial synthesis of fructose from inulin. This procedure requires the thermostable property of inulinase because it permits high-rate hydrolysis of inulin and prevents the huge microbial contamination that may occur if the process were carried out at ambient temperature [117]. Comparing it with the industrial inulinase Novozyme, Gill et al. discovered that the one produced by *Aspergillus fumigatus* (MTCC no. 3009) has a higher specific activity (23 IU/mg protein versus only 8 IU/mg protein), is active at higher temperatures and pH levels (60 °C versus 40 °C and 5.5 versus 4.7, respectively), and has a significantly higher residual activity (over 50% after two hours, whereas the industrial inulinase Novozyme only has 5.2% after two hours).

#### 2.3.2. The Effect of Metal Ions and Protein Inhibitors on Inulinase Activity

Some metal ions added to the reaction medium increase inulinase activity, while others inhibit it. *Kluyveromyces marxianus* produces inulinase, which is activated by Co^2+^, Mn^2+^, Mg^2+^, and low concentrations of SDS (0.001%) and inactivated by Cu^2+^, Fe^3+^, Zn^2+^, Tween 20, Tween 80, and Brij-35 [118], as well as Ca^2+^, Ba^2+^, Zn^2+^, and Na^+^.

Low concentrations of Ca^2+^, K^+^, Na^+^, Zn^2+^, and Cu^2+^ activate inulinase produced by *Cryptococcus aureus*, while Mg^2+^, Hg^2+^, and Ag^+^ inhibit it [58]. *Pichia guilliermondii* produces inulinase, which yields similar results [59]. Both yeast strains were isolated from marine environments.

The metal ions Hg^2+^ and Ag^+^ inhibit inulinase activity produced by fungi and bacteria, indicating the importance of amino acid residues with thiol groups in enzyme composition. The presence of these metals in the reaction medium inhibits the inulinases produced by *Arthrobacter* spp. [64], *Streptomyces* spp. [116], and *Aspergillus ochraceus* [65]. The effect of metal ions on inulinase activity is especially important when the substrate contains a high salt concentration. Pepstatin, EDTA, and 1,10-phenanthroline also inhibit inulinase activity [57,58], indicating that these enzymes are characterized as metallo-enzymes [59].

#### 2.3.3. Influence of Temperature on Inulinase Stability

Temperature significantly affects inulinase stability and activity. Maximal inulinase activity is frequently reported in the range of 52–64 °C, with a common optimum around 55–60 °C. At this temperature, the enzyme is stable and has a reasonable half-life (t_1/2_) for industrial processes (e.g., 137–173 min).

At temperatures above 60–70 °C, inulinase rapidly loses structural stability (denaturation). Studies have shown that after 60 min of incubation at 70 °C, the enzyme is completely inactivated. At 100 °C, inactivation occurs almost instantly.

Some studies have attempted to modify inulinases to function at lower temperatures, enhancing activity at 20–30 °C but decreasing stability at temperatures beyond 40 °C, despite the high optimum.

Due to its stability at 50–60 °C, inulinase is ideal for fructose syrup production processes, allowing for rapid reactions while preventing microbial contamination.

Because immobilization improves enzyme catalytic properties and allows for continuous reuse, enzymes are more economically viable in industrial applications [40,119]. Immobilized inulinases may exhibit higher thermal stability than free ones, occasionally maintaining their activity at higher temperatures due to the physical support.

## 3. Production and Purification Microbial Inulinase

Inulinases, one of the most important industrial enzymes, were first isolated from the roots of Jerusalem artichoke (*Helianthus tuberosus*) [19]. However, plant sources of inulinases cannot be commercially exploited due to low yield and an uneconomical process. Microorganisms are regarded as potential sources of inulinases because of their diversity, ease of cultivation, rapid growth, high enzyme yields, and ease of modification and management of growth and production conditions [53,120,121] when compared to animal and plant cells.

Important producers of inulinase are *Aspergillus* spp., *Penicillium* spp., *Kluyveromyces* spp., *Candida* spp., *Arthrobacter* spp., *Xanthomonas* spp., *Bacillus* spp., etc. Inulinases are produced both intracellularly and extracellularly. However, some microorganisms can produce the enzyme in both directions [53,54]. The majority of microorganisms produce either exo-inulinase or endo-inulinase, though a few microbial species produce both [36,107,122,123]. Some microbial strains, such as *Aspergillus niger* 12 [55] and *Kluyveromyces* sp. Y-85 [71], are known to produce both exo- and endo-inulinases intracellularly.

Isolating strains with higher inulinase productivity is important for ensuring practical and affordable methods because of demand that is increasing [124].

Inulinases can be produced through both submerged and solid-state fermentations with a variety of substrates and microorganisms [33,124,125,126].

Solid-state fermentation provides higher yields of inulinase compared to submerged fermentation [33,125]. Furthermore, in solid-state fermentation, low-cost agro-industrial wastes can be used as substrates for inulinase production, which will make the fermentation process more economically viable [62,115,125,127].

Enzyme purification in industrial biotechnology is a difficult process [45]. Regular purification methods need multiple chromatography gels and are expensive. An alternative technology might be required for this purpose. Aqueous two-phase systems (ATPSs) have been used as a liquid–liquid protein purification method for this purpose.

Aqueous two-phase systems (ATPSs) have been used for liquid–liquid protein purification. This selective method uses polyethylene glycol (PEG) and a salt at a critical concentration to create a liquid–liquid phase separation [128]. ATPS allows us to remove unwanted proteins and nucleic acids while also increasing enzyme yield and purity [124,129].

Besides being an optimal method of obtaining inulinases, their immobilization provides them with stability and has many additional advantages, including lower operating costs and the possibility to employ the immobilized biocatalyst repeatedly or continuously. Inulinases from various microbial sources have been immobilized in/on a variety of matrices/supports for use in applications such as batch and continuous high-fructose syrup (HFS) production.

Materials such as alginate [41,119], chitosan [42], and polyurethane [130] are used to immobilize enzymes.

Calcium alginate hydrogel is widely used for enzyme immobilization [41] due to its numerous benefits, including high porosity, ease of processing, and low cost. However, this material has some limitations related to biocompatibility and pore size [41]. To improve encapsulation efficiency and control enzyme release from the gel matrix, polymers (such as chitosan) have been covalently crosslinked, and the surface of alginate gel beads has been coated with other reagents (such as glutaraldehyde) [41].

Several studies have reported the repeated use of immobilized inulinase in successive batches [40,44,71,119,131].

The most important aspect of immobilization studies is the operational stability of the developed immobilized biocatalyst. The number of cycles used to test the immobilized biocatalyst reveals its operational and mechanical stability. Inulinase from *K. marxianus*, immobilized on Duolite A568, was successfully recycled in 55 batches to produce HFS, demonstrating the operational stability of the developed immobilized biocatalyst [71].

Despite the significant progress achieved in microbial inulinase production, several challenges continue to limit its large-scale industrial implementation. Enzyme yield remains highly dependent on the producing strain, fermentation conditions, and downstream processing efficiency, while purification procedures still account for a substantial proportion of the overall production costs. Furthermore, insufficient operational stability and limited enzyme reusability under industrial conditions remain important bottlenecks. Consequently, current research is increasingly focused on recombinant expression systems, synthetic biology approaches, protein engineering, and advanced immobilization strategies aimed at improving enzyme productivity, stability, and process scalability while reducing manufacturing costs.

Studies on the production of inulinases provide adequate knowledge about their purification techniques as well as a better understanding of their enzymatic behavior and characteristics. To increase the output of inulinase producers, we should focus on improving strains using recombinant technologies. The use of molecular techniques will also help achieve the desired biocatalytic properties. Future research should focus on highly efficient microbial species, strain improvement, and enzyme engineering.

Future developments in microbial inulinase production are expected to rely increasingly on integrated biotechnological approaches combining metabolic engineering, systems biology, artificial intelligence-assisted strain optimization, and sustainable bioprocess design. The identification of novel inulinase-producing microorganisms through metagenomic screening, together with advances in recombinant production platforms, will likely accelerate the development of highly efficient biocatalysts tailored for specific industrial and nutritional applications. Such innovations are expected to strengthen the role of microbial inulinases in the circular bioeconomy and the production of next-generation functional food ingredients.

## 4. Applications of Microbial Inulinases

It should be emphasized that the beneficial metabolic effects discussed in this section are primarily associated with the bioactive products generated through microbial inulinase activity, particularly fructo-oligosaccharides (FOSs) and other inulin-derived carbohydrates, rather than with the direct biological action of the enzyme itself. Therefore, microbial inulinases should be regarded as enabling biocatalysts that facilitate the production of functional ingredients capable of modulating gut microbiota composition and contributing indirectly to metabolic health.

**Figure 6:**
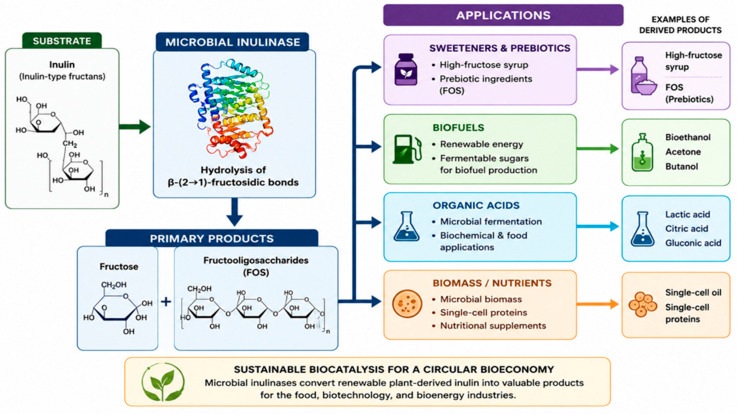
Schematic overview of the principal industrial and biotechnological applications of microbial inulinases. Through the enzymatic hydrolysis of inulin, microbial inulinases generate fructose and fructo-oligosaccharides (FOSs), which are subsequently utilized for the production of high-fructose syrup, prebiotic ingredients, biofuels (ethanol, acetone, and butanol), organic acids (lactic, citric, and gluconic acids), and microbial biomass, including single-cell proteins. The figure highlights the central role of microbial inulinases as biocatalysts linking renewable plant-derived substrates with value-added products for the food, biotechnology, and bioenergy industries.

Inulinases provide interesting perspectives; they can be used in a various applications including the food industry, bioethanol production, and pharmacology (Figure 6). Their primary biotechnological importance lies in catalyzing the hydrolysis of inulin into fructose and FOS, which are valuable intermediates for the production of functional food ingredients, nutraceuticals, biofuels, and other value-added bioproducts. Importantly, although microbial inulinases themselves are not generally used as therapeutic agents, they enable the production of bioactive compounds that have been associated with beneficial effects on metabolic health. Therefore, the contribution of microbial inulinases to disease prevention is predominantly indirect and results from the biological activities of their hydrolysis products rather than from direct pharmacological effects of the enzyme.

Using microbial inulinase, the enzymatic hydrolysis of inulin is accomplished in one step with a yield of 95% [21,33,57,132]. This advantage justifies research into optimizing the processes for obtaining inulinase and immobilizing the enzyme on various supports. Immobilization of the enzyme increases its stability and allows for multiple reuses in the inulin conversion process [40,119]. In addition, immobilization may improve enzyme resistance to pH and temperature variations, prolong operational activity, and facilitate downstream processing, making the overall biotechnological process more efficient and economically sustainable [133]. Consequently, immobilized inulinases are increasingly investigated for large-scale applications in the production of fructose syrups, fructooligosaccharides, biofuels, and other value-added bioproducts [120,133,134].

### 4.1. Potential Contribution to Metabolic and Nutritional Health

Metabolic disorders, including obesity, type 2 diabetes mellitus (T2DM), dyslipidemia, and metabolic syndrome, represent major global health challenges and are closely associated with chronic low-grade inflammation, insulin resistance, oxidative stress, and gut microbiota dysbiosis [132,133,134,135,136].

Microbial inulinases contribute to the prevention and management of these disorders primarily through their technological role as biocatalysts that convert inulin into fructose and short-chain fructo-oligosaccharides (FOSs). Current evidence indicates that the beneficial metabolic effects discussed in this section are mainly attributable to these enzymatic hydrolysis products and their downstream microbial metabolites rather than to direct biological activity of microbial inulinases themselves following ingestion.

FOS generated through microbial inulinase-mediated hydrolysis selectively stimulates the growth of beneficial intestinal microorganisms, particularly *Bifidobacterium* and *Lactobacillus species*, thereby promoting intestinal eubiosis and improving gut barrier integrity. Fermentation of FOS by the gut microbiota produces short-chain fatty acids (SCFAs), including acetate, propionate, and butyrate, which regulate glucose and lipid metabolism; reduce systemic inflammation; improve intestinal permeability; and stimulate secretion of glucagon-like peptide-1 (GLP-1) and peptide YY (PYY). Through these mechanisms, inulinase-generated products contribute indirectly to improved insulin sensitivity, enhanced satiety, reduced adiposity, and better metabolic homeostasis [2,137,138].

Furthermore, FOS has been associated with enhanced intestinal absorption of calcium and magnesium, contributing to bone health and nutritional status. Owing to their low caloric value and low glycemic index, these compounds are considered attractive functional food ingredients for dietary management of obesity, prediabetes, and T2DM [2,137]. Recent studies suggest that modulation of gut microbiota by inulin-derived oligosaccharides may reduce chronic low-grade inflammation associated with insulin resistance and obesity [15,135,137].

In addition, the principal contribution of microbial inulinases to metabolic health lies in enabling the industrial production of these bioactive carbohydrates rather than acting as therapeutic molecules themselves.

The use of inulinases represents an important biotechnological strategy for converting inulin into products that can help manage diabetes.

The resulting FOS and low-glycemic fructose exhibit a lower glycemic impact than sucrose and may improve postprandial glucose regulation by modulating gut microbiota composition, increasing SCFA production, and stimulating GLP-1 secretion [138].

It should be emphasized, however, that most clinical studies demonstrating improvements in glycemic control have evaluated supplementation with inulin-type fructans (ITFs) or FOS rather than oral administration of microbial inulinase itself. Clinical evidence suggests that approximately 10 g/day of ITFs consumed for at least 6–8 weeks may improve glycemic control in individuals with T2DM or prediabetes, although higher doses may induce gastrointestinal discomfort owing to rapid colonic fermentation [139].

In this context, nutritional strategies targeting gut microbiota modulation and metabolic balance have gained increasing attention. Microbial inulinase plays a crucial role in the degradation of inulin and fructans, oligosaccharides with prebiotic effects [13,92]. Consequently, microbial inulinases are increasingly investigated for their potential contribution to the development of functional foods and nutraceutical formulations targeting metabolic disorders. This has led to an increased interest in the health benefits of fructo-oligosaccharides (FOSs) [13,25,134].

A recent randomized, double-blind, placebo-controlled clinical trial evaluated microbial inulinase supplementation in healthy adults for four weeks. The study included men and women aged 20–60 with a BMI of 18.5–29.9 kg/m^2^ and a daily intake of two meals [140]. The enzyme used in this study, OPTIZIOME^®^ Inulinase (also marketed as OPTIZIOME^®^ Fructanase; BIO-CAT, Inc., Troy, VA, USA), is a non-genetically modified inulinase preparation containing a filtered enzyme concentrate obtained from *Aspergillus tubingensis* (reclassified from *Aspergillus niger* in 2022) and tapioca maltodextrin. Each inulinase capsule was formulated to contain 1000 INU. Placebo capsules contained 400 mg of tapioca maltodextrin. Participants in the inulinase group consumed 2000 INU per day (1000 INU per capsule). To ensure the study’s safety, clinical blood tests, haematology, lipid profile, high-sensitivity C-reactive protein, insulin, lactate, and uric acid were evaluated. Gastrointestinal symptoms were recorded weekly using the 15-item Gastrointestinal Symptom Rating Scale.

There were no clinically significant differences between the inulinase and placebo groups in terms of hematological or biochemical parameters, inflammatory markers, gastrointestinal symptoms, or adverse events, indicating that oral microbial inulinase is well tolerated in the conditions tested [140]. Microbial inulinase supplementation showed a good safety and tolerability profile in healthy adults. However, a dose-ranging trial in people with FODMAP, fructan, or inulin food sensitivity or irritable bowel syndrome is still required.

As the enzymes are susceptible to degradation in the acidic gastric environment and by intestinal proteases, efficient oral delivery of inulinase is difficult [141]. Therefore, incorporation of inulinase into liposomes represents a promising strategy to improve enzyme stability, protect against harsh gastrointestinal conditions, enhance bioavailability, and enable controlled release [142,143].

Moreover, liposomes may enhance the residence time of enzymes in the gastrointestinal tract and improve targeted delivery, thereby increasing the therapeutic potential of inulinase-based formulations [141,142,143].

Products generated through microbial inulinase-mediated hydrolysis of inulin may also contribute to improved lipid metabolism and cardiovascular health. FOSs and their fermentation products have been associated with reductions in serum LDL cholesterol and triglycerides, attenuation of hepatic lipogenesis, improvement of intestinal barrier integrity, and decreased metabolic endo-toxemia, thereby reducing chronic inflammation and cardiovascular risk [13,15,135,144].

Similarly, FOS generated by microbial inulinases may contribute to the modulation of obesity-related inflammation by stimulating the proliferation of beneficial bacteria (*Bifidobacterium* and *Akkermansia*) and the production of short-chain fatty acids (SCFAs) while also regulating glucose metabolism and inhibiting hepatic lipogenesis. At the same time, this enzyme promotes the production of metabolites that improve postprandial fat oxidation, thereby optimizing the efficient burning of lipids following a meal [138,145].

In the case of obesity, by facilitating the synthesis of metabolites that enhance fat oxidation and stimulate satiety hormones (such as GLP-1 and Peptide YY), inulinase-derived products optimize appetite control and effectively reduce body mass index, spontaneous caloric intake, and visceral fat [146]. Therefore, dietary supplementation with inulin-derived oligosaccharides may represent a promising complementary strategy for obesity prevention and long-term weight management.

Applying these enzymes to agro-industrial residues, like sisal root, shows significant potential for the long-term development of functional foods that directly contribute to combating type 2 diabetes and obesity [43]. Similarly, inulin-rich substrates such as Jerusalem artichoke and chicory roots may serve as sustainable raw materials for the large-scale production of bioactive oligosaccharides with applications in nutraceutical and functional food industries [13,43].

These metabolic effects highlight the important role of inulinase-derived compounds in the regulation of energy homeostasis and adipose tissue metabolism [138,145,146].

Overall, microbial inulinases represent promising biotechnological tools for the development of innovative dietary strategies aimed at preventing and managing metabolic and nutritional diseases through modulation of gut microbiota composition and metabolic homeostasis. Overall, current evidence indicates that microbial inulinases should be regarded primarily as enabling biocatalysts for the production of functional carbohydrates with demonstrated metabolic benefits. While oral microbial inulinase supplementation appears to be safe in healthy adults (Table 5), most evidence supporting prevention of obesity, T2DM, metabolic syndrome, and cardiovascular disorders is currently based on the biological effects of inulinase-generated FOS, fructose, and SCFAs rather than on the direct therapeutic actions of the enzyme itself [13,137,138,144]. Future clinical studies should distinguish between the metabolic effects of inulin-derived products and those of oral microbial inulinase supplementation.

### 4.2. Other Applications

Inulin is an attractive feedstock for both energy-based and product-based biorefinery systems. It can also be used as a feedstock to produce biofuels (ethanol and butanol) and biochemicals (organic acids and poly-γ-glutamate).

The superior properties of fructose over sucrose have increased the global demand for fructose. As a result, a simple and highly productive process for extracting fructose from inulin has emerged. Inulinase converts inulin directly to fructose in a single step, producing up to 95% fructose.

The literature contains numerous studies on the production of fructose from inulin and inulin-rich plant materials using both free and immobilized inulinase in batch and continuous systems [147,148].

Furthermore, Cardoso et al. demonstrated that *Aspergillus welwitschiae* is a promising source of enzymes that can convert agricultural waste into high-value-added products such as dietary sweeteners or even biofuels, offering a sustainable solution for biotechnology [149].

The production of bioethanol from diverse unconventional and economical material sources is garnering heightened interest among researchers. In this context, a new application for inulinases is the production of bioethanol. Biochemical and thermochemical conversion technologies can turn biomass into biofuels like biodiesel and other liquids. Sugar or starch from crops continues to be the primary feedstock for ethanol production worldwide, with 5% to 90% of the mixture being gasoline. Thus, inulin and inulin-containing plant materials have sparked interest in the use of inulinase to produce bioethanol [150]. As an example, a mixture of *A. niger* 817 and *S. cerevisiae* was used for saccharification and ethanol production from Jerusalem artichoke tubers [151]. The endo-inulinase gene from *Arthrobacter* sp. was inserted into the d sequence of *Saccharomyces* sp. to produce ethanol from Jerusalem artichoke tubers [28]. Genes encoding exo-inulinase from *Pichia guilliermondii* and *Penicillium janthinellum* were expressed in *Saccharomyces* sp. individually, and both were used for direct ethanol production from Jerusalem artichoke tubers [152,153].

Other applications of inulinases include the production of single-cell oil and single-cell proteins [151]. Thus, hydrolysates of Jerusalem artichoke tubers are reported as suitable substrates for the production of single-cell oil [154], and the marine yeast *C. aureus* has been used to produce unicellular proteins by culturing it on inulin hydrolysate from Jerusalem artichoke tubers [155].

Inulinase from *A. niger* has also been used for the production of citric acid [156]. Inulinases have also been used to produce 2,3-butanediol [157] and lactic acid [151]. A commercial preparation of inulinase, Fructozyme L, has been used for the production of tequila from the juice extracted from Agave tequilana [158].

## 5. Conclusions and Future Perspectives

The purpose of this review was to provide a comprehensive overview of microbial inulinases, multifunctional enzymes that hydrolyze inulin to produce prebiotic compounds, particularly fructo-oligosaccharides, which may contribute to dietary strategies aimed at supporting metabolic and nutritional health through modulation of the gut microbiota. Beyond their role in the production of prebiotic ingredients, microbial inulinases also have considerable biotechnological potential, contributing to the production of bioethanol, single-cell oils, single-cell proteins, citric acid, gluconic acid, lactic acid, butanol, sorbitol, and other value-added compounds.

The wide-ranging uses of inulinases definitively demonstrate their significance. Recent research has focused on developing efficient technologies for large-scale microbial inulinase production. Published papers on inulinases provide adequate information about purification techniques and properties, as well as data on reaction kinetics and enzymatic structure.

In order to increase the productivity of inulinase producers, more emphasis has been placed on strain improvement through recombinant technologies. Recently, there have been significant advances in the production and purification of microbial inulinase, with a focus on genetic engineering to improve enzyme production and functional properties.

Genetically modified microorganisms can yield elevated amounts of inulinase, hence decreasing the cost of inulin hydrolysis and enhancing its economic feasibility for industrial applications. By optimizing strains for increased inulinase production, biotechnological processes involving inulin can become more productive, efficient, and cost-effective, thereby contributing to the development of sustainable bioprocesses [159,160,161].

It is worth noting that, as demand for fossil fuels grows, their rapid depletion and negative impact on the environment make renewable energy sources an appealing and irreplaceable alternative. This results in increased focus on enhancing biofuel production technology, encompassing the advancement of more effective catalysts, the optimization of process parameters, and the utilization of previously underexploited raw materials. In this regard, microbial inulinases that biochemically convert agricultural waste rich in inulin into products facilitating bioethanol production will play an important part as functional enzymes that transform biomass into biofuels in the future.

Microbial inulinases continue to attract considerable scientific and industrial interest due to their versatility, substrate specificity, and broad applicability in food biotechnology, pharmaceutical production, and biorefinery processes. As highlighted throughout this review, advances in microbial screening, fermentation technologies, and enzyme characterization have significantly expanded the availability of inulinases with improved catalytic properties suitable for diverse industrial applications.

Beyond their established role in the production of high-fructose syrups and fructo-oligosaccharides (FOSs), microbial inulinases are increasingly recognized as enabling technologies for the development of functional foods. Although the health-promoting effects are primarily attributed to the prebiotic compounds generated by enzymatic hydrolysis rather than to the enzymes themselves, microbial inulinases play an essential role in the sustainable production of these bioactive ingredients. Consequently, they indirectly contribute to gut microbiota modulation, promoting the growth of beneficial microorganisms such as *Bifidobacterium* and *Lactobacillus* spp., which has been associated with improved metabolic homeostasis, immune regulation, and gastrointestinal health.

Recent research shows that supplementing the amount of kestose from FOS (FOS enriched with kestose >85% of kestose, compared to normal FOS—33% of kestose) can highlight the relationship between probiotics and other functional microorganisms in modulating the intestinal microbiota and strengthening the link between diet, nutrition, and health. This supplementation would improve intestinal populations of beneficial bacteria, particularly those from the *Firmicutes*, *Bacteroidetes*, and *Proteobacteria* phyla, which could help improve the symptoms of certain conditions, such as atopic dermatitis (AD). Kim et al. show that, in experiments on mice with induced AD, the group that was administered FOS enriched with kestose showed improvements in the intestinal microbiota, high levels of SCFA and suppression of Th2 (T helper) cell activation [162].

Of note is the research conducted by Nakaoka et al., which investigated the effect of administering 9 g of 1-kestose (a FOS) daily for 12 weeks to improve the prognosis of patients diagnosed with pancreatic ductal adenocarcinoma (PDAC) (as less than half of them did not respond to chemotherapy). Thus, the researchers observed that, in the group that was administered 1-kestose, the cancer marker CA19-9 significantly decreased, the neutrophil-to-lymphocyte ratio (NLR) was reduced, the albumin levels did not decrease, the C-reactive protein level was decreased, and there was also a significant decrease in the number of *Escherichia coli* cells, compared to the group that was not administered this type of FOS [163].

To elucidate the mechanisms by which 1-kestose supplementation affects lipid metabolism and the gut microbiome, Kuramitsu et al. analyzed blood components, liver gene expression, gut microbiota, and bile acid composition of the cecal contents of rats, concluding that 1-kestose intake modifies the gut microbiota (reducing the abundance of *Firmicutes* and increasing the abundance of *Bifidobacterium* and *Anaerostipes*) and, implicitly, bile acid metabolism in the cecum (positive correlation between the presence of *Bifidobacterium* and bile acids CA and CDCA), potentially positively influencing lipid metabolism in the host [164].

It can be said that research on FOSs is oriented both towards elucidating their role in the well-being of the body and towards industrial research aimed at producing FOSs that bring real benefits (constituting a nutritional source for beneficial gut bacteria) when ingested.

At the Future Food-Tech San Francisco 2026 conference, the South Korean company Samyang Corporation presented its high-purity soluble crystalline fiber Kestose in a technical presentation. Dr. Hyerim Kim, principal scientist at Samyang Corp., presented key insights into global dietary fiber trends, as well as the physicochemical properties, functional benefits, and category-specific application cases for crystalline Kestose.

Kestose is a type of fructo-oligosaccharide (FOS), a prebiotic ingredient that serves as a nutrient source for beneficial gut bacteria. Unlike conventional liquid or powdered dietary fiber ingredients, Kestose is distinguished by its crystalline form. The crystalline Kestose also offers distinct nutritional benefits. Containing over 99% fructo-oligosaccharides, it supports dietary fiber intake while providing approximately 30% of the sweetness of sugar, with a sugar content of only approximately 1% [162]. As a result, crystalline Kestose is well-suited for use in high-value, nutrition-focused products such as snack bars, yoghurt, protein beverages, and dietary supplements.

Recent research has shifted from simply identifying novel microbial producers toward optimizing enzyme performance through recombinant expression, protein engineering, immobilization technologies, and process intensification. These approaches aim to improve enzyme stability, catalytic efficiency, substrate specificity, and industrial scalability while reducing production costs. In parallel, advances in systems biology, metagenomics, and microbiome research are providing new opportunities to identify previously unexplored inulinase-producing microorganisms and to better understand the interactions between enzymatically generated prebiotics and the intestinal microbial ecosystem.

Future research should also focus on integrating microbial inulinases into precision nutrition strategies, personalized functional foods, and sustainable bioprocesses aligned with circular bioeconomy principles. Although numerous in vitro and experimental studies have demonstrated promising biological effects of FOS produced by microbial inulinases, additional clinical investigations are required to establish direct evidence linking specific enzymatic production processes with long-term health outcomes in humans.

Overall, microbial inulinases represent promising biotechnological tools for the development of innovative dietary strategies aimed at supporting metabolic and nutritional health through modulation of gut microbiota composition and metabolic homeostasis. Their continued development, together with advances in enzyme engineering, microbiome science, and translational biotechnology, is expected to further expand their contribution to functional foods and evidence-based nutritional strategies supporting metabolic and nutritional health.

## Figures and Tables

**Table 5:** Potential metabolic health benefits associated with bioactive compounds generated through microbial inulinase-mediated hydrolysis of inulin.

Inulinase-Derived Product	Mechanism of Action	Metabolic Benefits	Potential Clinical Relevance	References
Fructo-oligosaccharides (FOSs)	Selective stimulation of beneficial gut microbiota (*Bifidobacterium*, *Lactobacillus*)	Improvement of intestinal eubiosis and gut barrier integrity	Prevention of dysbiosis-associated metabolic disorders	[2,137,138]
Short-chain fatty acids (SCFAs) derived from FOS fermentation	Modulation of glucose and lipid metabolism; activation of G-protein-coupled receptors; anti-inflammatory effects	Improved insulin sensitivity and reduced systemic inflammation	Adjunctive management of T2DM and metabolic syndrome	[2,138]
SCFA-mediated satiety hormones (GLP-1 and PYY)	Increased secretion of satiety-related hormones following SCFA production	Reduced appetite and caloric intake	Obesity and weight management	[138,146]
Low-glycemic fructose derived from inulin hydrolysis	Lower postprandial glycemic response compared with sucrose	Stabilization of blood glucose levels	Dietary support in diabetes and prediabetes	[138,139]
Inulinase-derived metabolites involved in lipid regulation	Reduction of hepatic lipogenesis and improvement of lipid oxidation	Decreased serum triglycerides and LDL-C levels	Cardiovascular risk reduction	[144,145]
Inulinase-derived prebiotic compounds enhancing mineral absorption	Enhanced intestinal absorption of calcium and magnesium	Better bone mineralization and nutritional status	Prevention of mineral deficiencies	[2,137]
Antioxidant and anti-inflammatory metabolites generated from FOS fermentation	SCFA-mediated modulation of inflammatory and oxidative pathways	Protection against cellular oxidative damage	Prevention of diabetes-associated complications	[138]
Inulinase-derived compounds improving intestinal barrier integrity	Reduction of intestinal permeability and endotoxemia	Lower chronic low-grade inflammation	Prevention of cardiometabolic disorders	[144]
Liposomal inulinase formulations	Protection of enzyme against gastric degradation and controlled release	Increased enzyme stability and bioavailability	Development of oral nutraceutical and pharmaceutical formulations	[142,143]
Bioactive compounds obtained from agro-industrial residues	Bioconversion of inulin-rich biomass into bioactive compounds	Sustainable production of functional ingredients	Circular bioeconomy and functional food development	[43]

## Data Availability

No new data were created or analyzed in this study. Data sharing is not applicable to this article.

## References

[1] Gibson G.R., Hutkins R., Sanders M.E., Prescott S.L., Reimer R.A., Salminen S.J., Scott K., Stanton C., Swanson K.S., Cani P.D. (2017). Expert consensus document: The international scientific association for probiotics and prebiotics (ISAPP) consensus statement on the definition and scope of prebiotics. Nat. Rev. Gastroenterol. Hepatol..

[2] Hughes R.L., Alvarado D.A., Swanson K.S., Holscher H.D. (2022). The prebiotic potential of inulin-type fructans: A systematic review. Adv. Nutr..

[3] Slavin J. (2013). Fiber and prebiotics: Mechanisms and health benefits. Nutrients.

[4] Dahl W.J., Stewart M.L. (2015). Position of the academy of nutrition and dietetics: Health implications of dietary fiber. J. Acad. Nutr. Diet..

[5] Gargano D., Appanna R., Santonicola A., de Bartolomeis F., Stellato C., Cianferoni A., Casolaro V., Iovino P. (2021). Food allergy and intolerance: A narrative review on nutritional concerns. Nutrients.

[6] Hon E., Gupta S.K. (2021). Gastrointestinal Food Allergies and Intolerances. Gastroenterol. Clin. N. Am..

[7] Tuck C.J., Biesiekierski J.R., Schmid-Grendelmeier P., Pohl D. (2019). Food intolerances. Nutrients.

[8] Jyoti, Dey P. (2025). Mechanisms and implications of the gut microbial modulation of intestinal metabolic processes. NPJ Metab. Health Dis..

[9] Hazart D., Moulzir M., Delhomme B., Oheim M., Ricard C. (2025). Imaging the enteric nervous system. Front. Neuroanat..

[10] Guice J.L., Hollins M.D., Farmar J.G., Tinker K.M., Garvey S.M. (2023). Microbial inulinase promotes fructan hydrolysis under simulated gastric conditions. Front. Nutr..

[11] Ochoa K.C., Samant S., Liu A., Duysburg C., Marzorati M., Singh P., Hachuel D., Chey W., Wallach T. (2023). In Vitro Efficacy of Targeted Fermentable Oligosaccharides, Disaccharides, Monosaccharides, and Polyols Enzymatic Digestion in a High-Fidelity Simulated Gastrointestinal Environment. Gastro Hep Adv..

[12] Singh R.S., Chauhan K., Kennedy J.F. (2017). A panorama of bacterial inulinases: Production, purification, characterization and industrial applications. Int. J. Biol. Macromol..

[13] Singh R.S., Singh T., Hassan M., Kennedy J.F. (2020). Updates on inulinases: Structural aspects and biotechnological applications. Int. J. Biol. Macromol..

[14] Khosravi F., Fard E.M., Hosseininezhad M., Shoorideh H. (2023). Identification and characterization of inulinases by bioinformatics analysis of bacterial glycoside hydrolases family 32 (GH32). Eng. Life Sci..

[15] Sheng W., Ji G., Zhang L. (2023). Immunomodulatory effects of inulin and its intestinal metabolites. Front. Immunol..

[16] Kelly G. (2008). Inulin-type prebiotics–A review: Part 1. Altern. Med. Rev..

[17] Marteau P., Seksik P. (2004). Tolerance of probiotics and prebiotics. J. Clin. Gastroenterol..

[18] Fan Y.-G., Ning Y.-C., Chen J., Cao C.-Q., Wang H.-F., Han N.-F. (2024). Effects of inulin on the growth performance and tolerance in adverse environments of several probiotics. Res. Sq..

[19] Ramapriya R., Thirumurugan A., Sathishkumar T., Manimaran D.R. (2018). Partial purification and characterization of exo-inulinase produced from *Bacillus* sp.. J. Genet. Eng. Biotechnol..

[20] Neagu C., Bahrim G. (2011). Inulinases—A Versatile Tool for Biotechnology. Innov. Rom. Food Biotechnol..

[21] Ricca E., Calabro V., Curcio S., Iorio G. (2009). Fructose production by chicory inulin enzymatic hydrolysis: A kinetic study and reaction mechanism. Process. Biochem..

[22] Lima D.M., Fernandes P., Nascimento D.S., Ribeiro R.D.C.L.F., De Assis S.A. (2011). Fructose syrup: A biotechnology asset. Food Technol. Biotechnol..

[23] Maia O.B., Duarte R., Silva A.M. (2001). Evaluation of the components of a commercial probiotic in gnotobiotic mice experimentally challenged with *Salmonella enterica* subsp. Ser. Typhimurium. Vet. Microbiol..

[24] Kolida S., Tuohy K., Gibson G.R. (2002). Prebiotic effects of inulin and oligofructose. Br. J. Nutr..

[25] Miremadi F., Shah N.P. (2012). Applications of inulin and probiotics in health and nutrition. Int. Food Res. J..

[26] Chew W.S., Ng C.H., Tan D.J.H., Kong G., Lin C., Chin Y.H., Lim W.H., Huang D.Q., Quek J., Fu C.E. (2023). The global burden of metabolic disease: Data from 2000 to 2019. Cell Metab..

[27] Ge X.Y., Zhang W.G. (2005). Effects of octadecanoylsucrose derivatives on the production of inulinase by Aspergillus niger SL-09. World J. Microbiol. Biotechnol..

[28] Zhao C.H., Zhang T., Li M., Chi Z.M. (2010). Single cell oil production from hydrolysates of inulin and extract of tubers of Jerusalem artichoke by Rhodotorula mucilaginosa TJY15a. Process. Biochem..

[29] Liu X.Y., Chi Z., Liu G.L., Wang F., Madzak C., Chi Z.M. (2010). Inulin hydrolysis and citric acid production from inulin using the surfaceengineered *Yarrowia lipolytica* displaying inulinase. Metab. Eng..

[30] Dao T.H., Zhang J., Bao J. (2013). Characterization of inulin hydrolyzing enzyme(s) in commercial glucoamylases and its application in lactic acid production from Jerusalem artichoke tubers (Jat). Bioresour. Technol..

[31] Deepshikha, Verma P., Agrawal K. (2026). Fungal Inulinase: An insight into the unexplored dimension of multiproduct biorefineries. Biocatal. Agric. Biotechnol..

[32] Singh P.K., Kumar V., Yadav R., Shukla P. (2017). Bioengineering for Microbial Inulinases: Trends and Applications. Curr. Protein Pept. Sci..

[33] Singh R.S., Singh R.P., Pandey A., Negi S., Soccol C.R. (2017). 18—Inulinases. Current Developments in Biotechnology and Bioengineering: Production, Isolation and Purification of Industrial Products, Production, Isolation and Purification of Industrial Products.

[34] Hughes S.R., Qureshi N., López-Núnez J.C., Jones M.A., Jarodsky J.M., Galindo-Leva L.Á., Lindquist M.R. (2017). Utilization of Inulin-ContainingWaste in Industrial Fermentations to Produce Biofuels and Bio-Based Chemicals. World J. Microbiol. Biotechnol..

[35] Gao L., Chi Z., Sheng J., Wang L., Li J., Gong F. (2007). Inulinase-producing marine yeasts: Evaluation of their diversity and inulin hydrolysis by their crude enzymes. Microb. Ecol..

[36] Sheng J., Chi Z., Li J., Gao L., Gong F. (2007). Inulinase production by the marine yeast Cryptococcus aureus G7a and inulin hydrolysis by the crude inulinase. Process. Biochem..

[37] Alzain A.A., Almogaddam M.A., Yousif R., Alqarni M.H., Foudah A.I., Osman W., Elamin K.M., Mohamed H.M., Moglad E., Ashour A. (2025). Molecular Docking, Molecular Dynamics Simulation, and Pharmacophore-Based Virtual Screening Unveil Natural Compounds with TIM-3 Inhibitory Activity. J. Pharm. Bioallied Sci..

[38] Singh P.K., Shukla P. (2012). Molecular Modeling and Docking of Microbial Inulinases Towards Perceptive Enzyme–Substrate Interactions. Indian J. Microbiol..

[39] Basso A., Spizzo P., Ferrario V., Knapic L., Savko N., Braiuca P., Ebert C., Ricca E., Calabro V., Gardossi L. (2010). Endo- and exo-inulinases: Enzyme–substrate interaction and rational immobilization. Biotechnol. Prog..

[40] Mateo C., Palomo J.M., Fernandez-Lorente G., Guisan J.M., Fernandez-Lafuente R. (2007). Improvement of enzyme activity, stability and selectivity via immobilization techniques. Enzym. Microb. Technol..

[41] Zhou Z., Li G., Li Y. (2010). Immobilization of Saccharomyces cerevisiae alcohol dehydrogenase on hybrid alginate-chitosan beads. Int. J. Biol. Macromol..

[42] Yewale T., Singhal R.S., Vaidya A.A. (2013). Immobilization of inulinase from Aspergillus niger NCIM 945 on chitosan and its application in continuous inulin hydrolysis. Biocatal. Agric. Biotechnol..

[43] de Araujo Ribeiro G.C., Fernandes P., Silva D.A.A., Brandão H.N., de Assis S.A. (2021). Inulinase from Rhodotorula mucilaginosa: Immobilization and application in the production of fructooligosaccharides. Food Sci. Biotechnol..

[44] Eremia M.-C., Petrescu M.-M. (2021). Production, purification and immobilization of inulinases from *Aspergillius* species. Romanian Biotechnol. Lett..

[45] Germec M., Turhan I. (2020). Partial purification and characterization of Aspergillus niger inulinase produced from sugar-beet molasses in the shaking incubator and stirred-tank bioreactors. Int. J. Biol. Macromol..

[46] Muslim S.N., Zaki N.H., Hussein H.M. (2014). Purification and characterization of exoinulinase from Pseudomonas putida isolated from agricultural waste materials. Diyala J. Pure Sci..

[47] Henrissat B., Bairoch A. (1993). New families in the classification of glycosyl-hydrolases based on amino acid sequence similarities. Biochem. J..

[48] Henrissat B.A. (1991). Classification of glycosyl-hydrolases based on amino acid sequence similarities. Biochem. J..

[49] Pons T.O., Chinea O.G., Beldarraın A., Marquez G., Acosta N., Rodrıguez L., Valencia A. (1998). Structural model for family 32 of glycosyl-hydrolase enzymes. Proteins.

[50] Qin Y.-Q., Wang L.-Y., Yang X.-Y., Xu Y.-J., Fan G., Fan Y.-G., Ren J.-N., An Q., Li X. (2023). Inulin: Properties and health benefits. Food Funct..

[51] Bhanja A., Sutar P.P., Mishra M. (2022). Inulin-A polysaccharide:Review on its functionaland prebiotic efficacy. J. Food Biochem..

[52] Canazza E., Grauso M., Mihaylova D., Lante A. (2025). Techno-Functional Properties and Applications of Inulin in Food Systems. Gels.

[53] Singh R.S., Singh R.P. (2010). Production of fructooligosaccharides from inulin by endoinulinases and their prebiotic potential. Food Technol. Biotechnol..

[54] Zhang Y., Liu R., Song B., Li L., Sh R., Ma X., Zhang L., Li X. (2024). Recent advances in inulin polysaccharides research: Extraction, purification, structure, and bioactivities. Chem. Biol. Technol. Agric..

[55] Chen H.Q., Chen X.M., Li Y., Wang J., Jin Z.Y., Xu X.M., Zhao J.W., Chen T.X., Xie Z.J. (2009). Purification and characterization of exo- and endo-inulinase from Aspergillus ficuum JNSP5-06. Food Chem..

[56] El Attar N.A. (2011). Studies on Possible Activation of Microbial Inulinase Production Using Gamma Radiation Under Solid State Fermentation. Bachelor’s Thesis.

[57] Chi Z., Chi Z., Zhang T., Liu G., Yue L. (2009). Inulinase-expressing microorganisms and applications of inulinases. Appl. Microbiol. Biotechnol..

[58] Sheng J., Chi Z., Gong F., Li J. (2008). Purification and characterization of extracellular inulinase from a marine yeast Cryptococcus aureus G7a and inulin hydrolysis by the purified Inulinase. Appl. Biochem. Biotechnol..

[59] Gong F., Zhang T., Chi Z., Sheng J., Li J., Wang X. (2008). Purification and characterization of extracellular inulinase from a marine yeast Pichia guilliermondii and inulin hydrolysis by the purified inulinase. Biotechnol. Bioprocess Eng..

[60] Khan N.T. (2022). Kluyveromyces fragilis in Food Biotechnology. J. Microbiol. Bacteriol. Res..

[61] Artyukhov V.G., Kovaleva T.A., Kholyavka M.G., Bityutskaya L.A., Grechkina M.V., Obraztsova T.B. (2009). Study of the oligomeric structure and some physicochemical properties of inulinase from Kluyveromyces marxianus Y-303. Biophysics.

[62] Yupanqui-Mendoza S.L., de Arruda P.V., Castelo da Silva G.M. (2022). Statistical sequential optimization of process parameters for inulinase production by Kluyveromyces marxianus ATCC 36907 in solid-state fermentation using beer residue. Biocatal. Agric. Biotechnol..

[63] Fonseca G.G., Heinzle E., Wittmann C., Gombert A.K. (2008). The yeast Kluyveromyces marxianus and its biotechnological potential. Appl. Microbiol. Biotechnol..

[64] Kassem M.M., Hauka F.I.A., Afify A.H., El-Sawah A.M. (2013). Production and properties of inulinase from *Arthrobacter* sp.. J. Agric. Chem. Biotechnol..

[65] Guimaraes L.H.S., Terenzi H.F., Polizeli M.L., Jorge J.A. (2007). Production and characterization of a thermostable extracellular β-Dfructofuranosidase produced by Aspergillus ochraceus with agro-industrial residues as carbon sources. Enzym. Microb. Technol..

[66] Akimoto H., Kiyota N., Kushima T., Nakamura T., Ohta K. (2000). Molecular cloning and sequence analysis of an endo-inulinase gene from Penicillium sp. strain TN-88. Biosci. Biotechnol. Biochem..

[67] Pessoni R.A., Braga M.R., Figueiredo-Ribeiro R.C. (2007). Purification and properties of exo-inulinases from *Penicillium janczewskii* growing on distinct carbon sources. Mycologia.

[68] Neera N., Ramana V.K., Gopala N., Sharma R.K. (2018). Production of Inulinase by Fusarium sp. and its Application for Fructo-oligosaccharide Production for use as Prebiotics. Def. Life Sci. J..

[69] Yuan B., Hu N., Sun J., Wang S.A., Li F.L. (2012). Purification and characterization of a novel extracellular inulinase from a new yeast species *Candida kutaonensis* sp. nov. *KRF1(T). Appl. Microbiol. Biotechnol..

[70] Wei W., Wang S., Zhu X., Wan W. (1999). Isolation of a mutant of Kluyveromyces sp. Y-85 resistant to catabolite repression. J. Biosci. Bioeng..

[71] Singh R.S., Dhaliwal R., Puri M. (2007). Production of high fructose syrup from Asparagus inulin using immobilized exoinulinase from *Kluyveromyces marxianus* YS-1. J. Ind. Microbiol. Biotechnol..

[72] Kushi R.T., Monti R., Contiero J. (2000). Production, purification and characterization of an extracellular inulinase from *Kluyveromyces marxianus* var. bulgaricus. J. Ind. Microbiol. Biotechnol..

[73] Wang S.-A., Li F.-L. (2013). Invertase SUC2 Is the Key Hydrolase for Inulin Degradation in *Saccharomyces cerevisiae*. Appl. Environ. Microbiol..

[74] Li Y., Liua G.L., Wang K., Chi Z.M., Madzaka C. (2012). Overexpression of the endo-inulinase gene from *Arthrobacter* sp. S37 in *Yarrowia lipolytica* and characterization of the recombinant endo-inulinase. J. Mol. Catal. B Enzym..

[75] Shen J., Zhang R., Li J., Tang X., Li R., Wang M., Huang Z., Zhou J. (2015). Characterization of an exo-inulinase from Arthrobacter: A novel NaCl-tolerant exo-inulinase with high molecular mass. Bioengineered.

[76] Raba’atun Adawiyah S., Shuhaimi M., Yazid A.M., Manaf A., Rosli N., Sreeramanan S. (2011). Molecular cloning and sequence analysis of an inulinase gene from an *Aspergillus* sp.. World J. Microbiol. Biotechnol..

[77] Dotsenko A., Rozhkova A., Denisenko J., Shashkov I., Sinitsyn A. (2023). Stabilization of elements of secondary structure in *Aspergillus awamori* exo-inulinase for thermostability improvement. Bioresour. Technol. Rep..

[78] Arand M., Golubev A.M., Neto J.R., Polikarpov I., Wattiez R., Korneeva O.S., Eneyskaya E.V., Kulminskaya A.A., Shabalin K.A., Shishliannikov S.M. (2002). Purification, characteriza- tion, gene cloning and preliminary X-ray data of the exo-inulinase from *Aspergillus awamori*. Biochem. J..

[79] Mutanda T., Wilhelmi B., Whiteley C.G. (2009). Controlled production of fructose by an exoinulinase from *Aspergillus ficuum*. Appl. Biochem. Biotechnol..

[80] Pouyez J., Mayard A., Vandamme A.M., Roussel G., Perpete E.A., Wouters J., Housen I., Michaux C. (2012). First crystal structure of an endo-inulinase, inu2, from Aspergillus ficuum: Discovery of an extra-pocket in the catalytic domain responsible for its endo-activity. Biochimie.

[81] Mutanda T., Wilhelmi B., Whiteley C.G. (2008). Response surface methodology: Synthesis of inulooligosaccharides with an endoinulinase from *Aspergillus niger*. Enzym. Microb. Techol..

[82] Moriyama S., Tanaka H., Uwataki M., Muguruma M., Ohta K. (2003). Molecular cloning and characterization of an exoinulinase gene from *Aspergillus niger* strain 12 and its expression in *Pichia pastoris*. J. Biosci. Bioeng..

[83] Coitinho J.B., Guimaraes V.M., de Almeida M.N., Falkoski D.L., de Queiroz J.H., de Rezende S.T. (2010). Characterization of an exoinulinase produced by *Aspergillus terreus* CCT 4083 grown on sugar cane bagasse. J. Agric. Food Chem..

[84] Beroigui O., El Ghadraoui L., Errachidi F. (2023). Production, purification, and characterization of inulinase from *Streptomyces anulatus*. J. Basic Microbiol..

[85] Cho Y.J., Yun J.W. (2002). Purification and characterization of endoinulinase from *Xanthomonas oryzae* No.5. Proc. Biochem..

[86] Lima M.Z.T., Muniz J.R.C. (2019). Image from the RCSB PDB (rcsb.org) of PDB ID 6NUN (Lima, M.Z.T., Muniz, J.R.C.) Structure of GH32 Hydrolase from *Bifidobacterium adolescentis* in Complex with Fructose. https://pdbj.org/mine/summary/6nun.

[87] Nagem R.A.P., Rojas A.L., Golubev A.M., Korneeva O.S., Eneyskaya E.V., Kulminskaya K.N., Neustroev K.N., Polikarpov I. (2004). Crystal structure of exo-inulinase from Aspergillus awamori: The enzyme fold and structural determinants of substrate recognition. J. Mol. Biol..

[88] Holyavka M.G., Artyukhov V.G., Makin S.M. (2015). Investigation of inulinase permolecular organization from producers of the genus *Aspergillus* using several computing and experimental methods. Biophysics.

[89] Holyavka M., Artyukhov V., Kovaleva T. (2016). Structural and functional properties of inulinases: A review. Biocatal. Biotransformation.

[90] Liu G.-L., Chi Z., Chi Z.-M. (2013). Molecular characterization and expression of microbial inulinase genes. Crit. Rev. Microb..

[91] Jochen R., Willmitzer L., Heyer A.G. (1998). Production of 1-Kestose in Transgenic Yeast Expressing a Fructosyltransferase from *Aspergillus foetidus*. J. Bacteriol..

[92] Fawzi E. (2011). Comparative Study of Two Purified Inulinases from Thermophile Thielavia terrestris NRRL 8126 and Mesophile *Aspergillus foetidus* NRRL 337 Grown on *Cichorium intybus*. Braz. J. Microbiol..

[93] Singh R.S., Singh T., Kennedy J.F. (2021). Understanding the interactive influence of hydrolytic conditions on biocatalytic production of fructooligosaccharides from inulin. Int. J. Biol. Macromol..

[94] Van den Ende W. (2018). Novel fructan exohydrolase: Unique properties and applications for human health. J. Exp. Bot..

[95] Park S., Han Y., Kim H., Song S., Uhm T.-B., Chae K.-S. (2003). Trp17 and Glu20 residues in conserved WMN(D/E)PN motif are essential for *Aspergillus ficuum* endo-inulinase (EC 3.2.1.7) activity. Biochemistry.

[96] Ma J., Li T., Tan H., Liu W., Yin H. (2020). The Important Roles Played in Substrate Binding of Aromatic Amino Acids in Exo-Inulinase From *Kluyveromyces cicerisporus* CBS 4857. Front. Mol. Biosci..

[97] Chi Z., Chi Z., Zhang T., Liu G., Li J., Wang X. (2009). Production, characterization and gene cloning of the extracellular enzymes from the marine-derived yeasts and their potential applications. Biotechnol. Adv..

[98] Zhang L., Zhao C., Wang J., Ohta Y., Wang Y. (2005). Inhibition of glucose on an exoinulinase from *Kluyveromyces marxianus* expressed in *Pichia pastoris*. Process. Biochem..

[99] Zhang T., Gong F., Chi Z., Liu G.L., Chi Z.M., Sheng J., Li J., Wang X.H. (2009). Cloning and characterization of the inulinase gene from a marine yeast *Pichia guilliermondii* and its expression in *Pichia pastoris*. Antonie Leeuwenhoek.

[100] Kim K.Y., Nascimento A.S., Golubev A.M., Polikarpov I., Kim C.S., Kang S., Kim S. (2008). Catalytic mechanism of inulinase from *Arthrobacter* sp. S37. Biochem. Biophys. Res. Commun..

[101] Dotsenko A., Denisenko J., Zorov I., Wasserman L., Semenova M., Korolev A., Rozhkova A., Sinitsyn A. (2023). Single substitution in α-helix of active center enhanced thermostability of *Aspergillus awamori* exo-inulinase. J. Mol. Graph. Model..

[102] Onodera S., Murakami T., Ito H., Mori H., Matsui H., Honma M., Chiba S., Shiomi N. (1996). Molecular cloning and nucleotide sequences of cDNA and gene encoding endo-inulinase from *Penicillium purpurogenum*. Biosci. Biotechnol. Biochem..

[103] Perez J.A., Rodriguez J., Rodriguez L., Ruiz T. (1996). Cloning and sequence analysis of the invertase gene INV1 from the yeast *Pichia anomala*. Curr. Genet..

[104] Meng G., Futterer K. (2003). Structural framework of fructosyl transfer in *Bacillus subtilis* levansucrase. Nat. Struct. Biol..

[105] Kwon H.J., Jeon S.J., You D.J., Kim K.H., Jeong Y.K., Kim Y.H., Kim Y.M., Kim B.W. (2003). Cloning and characterization of an exoinulinase from *Bacillus polymyxa*. Biotechnol. Lett..

[106] Zhou J., Lu Q., Peng M., Zhang R., Mo M., Tang X., Li J., Xu B., Ding J., Huang Z. (2015). Cold-active and NaCl-tolerant exo-inulinase from a cold-adapted *Arthrobacter* sp. MN8 and its potential for use in the production of fructose at low temperatures. J. Biosci. Bioeng..

[107] Nakamura T., Kuramori K., Zaita N., Akimoto H., Ohta K. (2001). Purification and properties of intracellular exo- and endo-inulinases from *Aspergillus niger* strain 12. Bulletin of the Faculty of Agriculture.

[108] Kang S., Kim S. (1999). Molecular cloning and sequence analysis of an endo-inulinase gene from *Arthrobacter* sp.. Biotechnol. Lett..

[109] Vijayaraghavan K., Yamini D., Ambika V., Sowdamini N.S. (2009). Trends in inulinase production—A review. Crit. Rev. Biotechnol..

[110] Vallejo-García L.C., Rodríguez-Alegría M.E., Munguía A.L. (2019). Enzymatic Process Yielding a Diversity of Inulin-Type Microbial Fructooligosaccharides. J. Agric. Food Chem..

[111] Mohamed S.A., Salah H.A., Moharam M.E., Foda M.S., Fahmy A.S. (2015). Characterization of two thermostable inulinases from *Rhizopus oligosporus* NRRL 2710. J. Genet. Eng. Biotechnol..

[112] Olshannikova S.S., Malykhina N.V., Lavlinskaya M.S., Sorokin A.V., Yudin N.E., Vyshkvorkina Y.M., Lukin A.N., Holyavka M.G., Artyukhov V.G. (2022). Novel Immobilized Biocatalysts Based on Cysteine Proteases Bound to 2-(4-Acetamido-2-sulfanilamide) Chitosan and Research on Their Structural Features. Polymers.

[113] Trivedi S., Divecha J., Shah T., Shah A. (2015). Rapid and efficient bioconversion of chicory inulin to fructose by immobilized thermostable inulinase from *Aspergillus tubingensis* CR16. Bioresour. Bioprocess..

[114] Zherebtsov N.A., Shelamova S.A., Abramova I.N. (2002). Biosynthesis of inulinases by *Bacillus* bacteria. Appl. Biochem. Microbiol..

[115] Mazutti M., Ceni G., Luccio M., Treichel H. (2007). Production of inulinase by solid-state fermentation: Effect of process parameters on production and preliminary characterization of enzyme preparations. Bioprocess Biosyst. Eng..

[116] Sguarezi C., Longo C., Ceni G., Boni G., Silva M.F., Luccio M., Mazutti M.A., Maugeri F., Sharma A.D., Kainth S. (2006). Inulinase production using garlic (*Allium sativum*) powder as a potential substrate in *Streptomyces* sp.. J. Food Engin..

[117] Gill P.K., Manhas R.K., Singh P. (2006). Comparative analysis of thermostability of extracellular inulinase activity from *Aspergillus fumigates* with commercially available (Novozyme) inulinase. Bioresour. Technol..

[118] Singh R.S., Bhermi H.K. (2008). Production of extracellular exoinulinase from *Kluyveromyces marxianus* YS-1 using root tubers of *Asparagus officinalis*. Bioresour. Technol..

[119] Rehman H.U., Aman A., Silipo A., Qader S.A.U., Molinaro A., Ansari A. (2013). Degradation of complex carbohydrate: Immobilization of pectinase from *Bacillus licheniformis* KIBGE-IB 21 using calcium alginate as a support. Food Chem..

[120] Kango N., Jain S.C. (2011). Production and properties of microbial inulinases: Recent advances. Food Biotechnol..

[121] Das D., Selvaraj R., Bhat M.R. (2019). Optimization of inulinase production by a newly isolated strain *Aspergillus flavus* var flavus by solid state fermentation of *Saccharum arundinaceum*. Biocatal. Agric. Biotechnol..

[122] Barthomeuf C., Regerat F., Pourrat H. (1991). Production of inulinase by a new mold of *Penicillum rugulosum*. J. Ferment. Bioeng..

[123] Dinarvand M., Ariff A.B., Moeini H., Masomian M., Mousavi S.S., Nahavandi R., Mustafa S. (2012). Effect of extrinsic and intrinsic parameters on inulinase production by *Aspergillus niger* ATCC 20611. Electron. J. Biotechnol..

[124] Abo Elsoud M.M., Mouafi F.E., Elgamal N.N., Moharam M.E. (2023). Optimization of production and partial purification of inulinase from *Bacillus subtilis*. Biocatal. Agric. Biotechnol..

[125] Mazutti M.A., Skrowonski A., Boni G., Zabot G.L., Silva M.F., de Oliveira D., Di Luccio M., Filho F.M., Rodrigues M.I., Treichel H. (2009). Partial characterization of inulinases obtained by submerged and solid-state fermentation using agroindustrial residues as substrates: A comparative study. Appl. Biochem. Biotechnol..

[126] Chen H.Q., Chen X.M., Chen T.X., Xu X.M., Jin Z.Y. (2011). Extraction optimization of inulinase obtained by solid state fermentation of Aspergillus ficuum JNSP5-06. Carbohydr. Polym..

[127] Canatar M., Tufan H.N.G., Ünsal S.B.E., Koc C.Y., Ozcan A., Kucuk G., Basmak S., Yatmaz E., Germec M., Yavuz I. (2023). Inulinase and fructooligosaccharide production from carob using *Aspergillus niger* A42 (ATCC 204447) under solid-state fermentation conditions. Int. J. Biol. Macromol..

[128] Raghavarao K.S.M.S., Rastogi N.K., Gowthaman M.K., Karanth N.G. (1995). Aqueous two- phase extraction for downstream processing of enzymes, proteins. Adv. Appl. Microbiol..

[129] Klomklao S., Benjakul S., Visessanguan W., Simpson B.K., Kishimura H. (2005). Partitioning and recovery of proteinase from tuna spleen by aqueous two-phase systems. Process. Biochem..

[130] Marceli F.S., Rigo D., Mossi V., Dallago R.M., Henrick P., de Oliveira Kuhn G., Dalla Rosa C., Oliveira D., Oliveira J.V., Treichel H. (2013). Evaluation of enzymatic activity of commercial inulinase from *Aspergillus niger* immobilized in polyurethane foam. Food Bioprod. Process..

[131] Santa G.L.M., Bernardino S.M.S.A., Magalhães S., Mendes V., Marques M.P.C., Fonseca L.P., Fernandes P. (2011). From inulin to fructose syrups using sol-gel immobilized inulinase. Appl. Biochem. Biotechnol..

[132] Singh R.S., Singh T. (2019). Microbial inulinases and pullulanases in the food industry. Microbial Enzymes and Additives for the Food Industry.

[133] Silva M.F., Rigo D., Mossi V., Golunski S., Kuhn G.O., Luccio M., Dallago R., de Oliveira D., Oliveira J.V., Treichel H. (2013). Enzymatic synthesis of fructooligosaccharides by inulinases from *Aspergillus niger* and *Kluyveromyces marxianus* NRRL Y-7571 in aqueous–organic medium. Food Chem..

[134] WHO (2024). Global Report on Diabetes.

[135] Fahed G., Aoun L., Zerdan M.B., Allam S., Bouferraa Y., Assi H.I. (2022). Metabolic Syndrome: Updates on Pathophysiology and Management in 2021. Int. J. Mol. Sci..

[136] Turnbaugh P.J., Ley R.E., Hamady M., Fraser-Liggett C.M., Knight R., Gordon J.I. (2007). The human microbiome project. Nature.

[137] Green M., Arora K., Prakash S. (2020). Microbial medicine: Prebiotic and probiotic functional foods to target obesity and metabolic syndrome. Int. J. Mol. Sci..

[138] Dehghan P., Gargari B.P., Asgharijafarabadi M. (2013). Effects of high performance inulin supplementation on glycemic status and lipid profile in women with type 2 diabetes: A randomized, placebo-controlled clinical trial. Health Promot. Perspect..

[139] Wang L., Yang H., Huang H., Zhang C., Zuo H.X., Xu P., Niu Y.M., Wu S.S. (2019). Inulin-type fructans supplementation improves glycemic control for the prediabetes and type 2 diabetes populations: Results from a GRADE-assessed systematic review and dose-response meta-analysis of 33 randomized controlled trials. J. Transl. Med..

[140] Garvey S.M., LeMoire A., Wang J., Lin L., Sharif B., Bier A., Boyd R.C., Baisley J. (2024). Safety and Tolerability of Microbial Inulinase Supplementation in Healthy Adults: A Randomized, Placebo-Controlled Trial. Gastro Hep Adv..

[141] Wehaidy H.R. (2023). An Overview of Some Enzyme Stabilization Strategies: Advantages and Drawbacks. Egypt. J. Chem..

[142] Bozzuto G., Molinari A. (2014). Liposomes as nanomedical devices. Int. J. Nanomed..

[143] Nsairat H., Khater D., Sayed U., Odeh F., Al Bawab A., Alshaer W. (2022). Liposomes: Structure, composition, types, and clinical applications. Heliyon.

[144] Talukdar J.R., Cooper M., Lyutvyn L., Zeraatkar D., Ali R., Berbrier R., Janes S., Ha V., Darling P.B., Xue M. (2024). The effects of inulin-type fructans on cardiovascular disease risk factors: Systematic review and meta-analysis of randomized controlled trials. Am. J. Clin. Nutr..

[145] van der Beek C.M., Canfora E.E., Kip A.M., Gorissen S.H.M., Olde Damink S.W.M., van Eijk H.M., Holst J.J., Blaak E.E., Dejong C.H.C., Lenaerts K. (2018). The prebiotic inulin improves substrate metabolism and promotes short-chain fatty acid production in overweight to obese men. Metabolism.

[146] Nakajima H., Nakanishi N., Miyoshi T., Okamura T., Hashimoto Y., Senmaru T., Majima S., Ushigome E., Asano M., Yamaguchi M. (2022). Inulin reduces visceral adipose tissue mass and improves glucose tolerance through altering gut metabolites. Nutr. Metab..

[147] Singh R.S., Chauhan K., Singh R.P., Gahlawat S.K., Salar R.K., Siwach P., Duhan J.S., Kumar S., Kaur P. (2017). Enzymatic approaches for the synthesis of high fructose syrup. Plant Biotechnology: Recent Advancements and Developments.

[148] Qiu Y., Zhang Y., Zhu Y., Sha Y., Xu Z., Feng X., Li S., Xu H. (2019). Improving poly-(γ-glutamic acid) production from a glutamic acid-independent strain from inulin substrate by consolidated bioprocessing. Bioprocess Biosyst. Eng..

[149] Cardoso E.S., Martins N.Q., Azevedo R.A., Palmeira L.S., Quintanilha-Peixoto G., Andrade B., Santos M.P., Uetanabaro A.P., Silva E.G., Góes-Neto A. (2025). Production and application of inulinase by new isolates of *Aspergillus welwitschiae* from fermented peach-palm waste for the production of fructooligosaccharides. Food Chem..

[150] Li Y.F., Jiang H., Hu Z., Liu G.L., Chi Z.M., Chi Z. (2018). Overexpression of an inulinase gene in an oleaginous yeast, *Aureobasidium melanogenum* P10, for efficient lipid production from inulin. J. Mol. Microbiol. Biotechnol..

[151] Wang Z.P., Fu W.J., Xu H.M., Chi Z.M. (2014). Direct conversion of inulin into cell lipid by an inulinase-producing yeast *Rhodosporidium toruloides* 2F5. Bioresour. Technol..

[152] Shi T.Q., Huang H., Kerkhoven E.J., Ji X.J. (2018). Advancing metabolic engineering of *Yarrowia lipolytica* using the CRISPR/Cas system. Appl. Microbiol. Biotechnol..

[153] Zhao C.H., Cui W., Liu X.Y., Chi Z.M., Madzak C. (2010). Expression of inulinase gene in the oleaginous yeast *Yarrowia lipolytica* and single cell oil production from inulin containing materials. Metab. Eng..

[154] Saha B.C. (2006). Production of mannitol from inulin by simultaneous enzymatic saccharification and fermentation with *Lactobacillus intermedius* NRRL B-3693. Enzym. Microb. Technol..

[155] Xia J., Xu J., Liu X., Xu J., Wang X., Li X. (2017). Economic co-production of poly (malic acid) and pullulan from Jerusalem artichoke tuber by *Aureobasidium pullulans* HA-4D. BMC Biotechnol..

[156] Fages J., Mulard D., Rouquet J.J., Wilhelm J.L. (1986). 2, 3-Butanediol production from Jerusalem artichoke, *Helianthus tuberosus*, by *Bacillus polymyxa* ATCC 12 321 optimization of kLa profile. Appl. Microbiol. Biotechnol..

[157] Sun L.H., Wang X.D., Dai J.Y., Xiu Z.L. (2009). Microbial production of 2, 3-butanediol from Jerusalem artichoke tubers by *Klebsiella pneumonia*. Appl. Microbiol. Biotechnol..

[158] Li D., Dai J.Y., Xiu Z.L. (2010). A novel strategy for integrated utilization of Jerusalem artichoke stalk and tuber for production of 2, 3-butanediol by *Klebsiella pneumonia*. Bioresour. Technol..

[159] Mignogna D., Szabó M., Ceci P., Avino P. (2024). Biomass Energy and Biofuels: Perspective, Potentials, and Challenges in the Energy Transition. Sustainability.

[160] González-Gloria K.D., Tomás-Pejó E., Amaya-Delgado L., Rodríguez-Jasso R.M., Loredo-Trevino A., Singh A., Hans M., Martín C., Kumar S., Ruiz H.A. (2024). Biochemical and Biorefinery Platform for Second-Generation Bioethanol: Fermentative Strategies and Microorganisms. Fermentation.

[161] Tsigoriyna L., Arsov A., Gergov E., Petrova P., Petrov K. (2023). Influence of pH on Inulin Conversion to 2,3-Butanediol by *Bacillus licheniformis* 24: A Gene Expression Assay. Int. J. Mol. Sci..

[162] Kim J.H., Baek J., Sa S., Park J., Kih M., Kim W. (2021). Kestose-enriched fructo-oligosaccharide alleviates atopic dermatitis by modulating the gut microbiome and immune response. J. Funct. Foods.

[163] Nakaoka K., Ohno E., Kuramitsu K., Kuzuya T., Funasaka K., Tochio T., Fujii T., Takahashi H., Kondo N., Miyahara R. (2024). Efficacy of 1-Kestose supplementation in patients with pancreatic ductal adenocarcinoma: A randomized controlled pilot study. Nutrients.

[164] Kuramitsu K., Kubo M., Cindy F., Shibata T., Kadota Y., Kitaura Y. (2025). Effect of 1-Kestose on lipid metabolism in a high-fat-diet rat model. Nutrients.

